# Fungal Richness of *Cytospora* Species Associated with Willow Canker Disease in China

**DOI:** 10.3390/jof8040377

**Published:** 2022-04-07

**Authors:** Lu Lin, Meng Pan, Chengming Tian, Xinlei Fan

**Affiliations:** The Key Laboratory for Silviculture, Beijing Forestry University, Conservation of Ministry of Education, Beijing 100083, China; lulin0677@bjfu.edu.cn (L.L.); panmeng@bjfu.edu.cn (M.P.); chengmt@bjfu.edu.cn (C.T.)

**Keywords:** biogeographic distribution, Diaporthales, new species, pathogens, phylogeny, *Salix*

## Abstract

Species of *Cytospora* are considered important plant pathogens of a wide range of plant hosts, especially Salicaceae plants. *Salix* (Salicaceae, Malpighiales) has been widely cultivated in China because of its strong ecological adaptability, fast growth, and easy reproduction. In this study, a total of eight species of *Cytospora* were discovered on *Salix* in China, including *C. ailanthicola*, *C. alba*, *C. chrysosperma*, *C. gigaspora*, *C. nivea*, *C. paracinnamomea*, *C. rostrata*, and *C. sophoriopsis*. Among them, *C. alba* and *C. paracinnamomea* were identified as novel species based on morphology and phylogenetic analyses of ITS, *act*, *rpb2*, *tef1-α*, and *tub2* gene sequences and were confirmed as pathogens of willow canker disease by pathogenicity tests. The mycelial growth rates of strains from these two novel species (*C. alba* and *C. paracinnamomea*) had optimum temperatures of 21 to 22 °C and an optimum pH value of 5 to 6. The effectiveness of six carbon sources on the mycelial growth showed that fructose and maltose had the highest influence. *Cytospora* species richness was significantly positively correlated with dry and wet areas. This study represents a significant evaluation of *Cytospora* associated with willow canker disease in China and provides a theoretical basis for predicting the potential risk of willow canker disease.

## 1. Introduction

**Figure 1:**
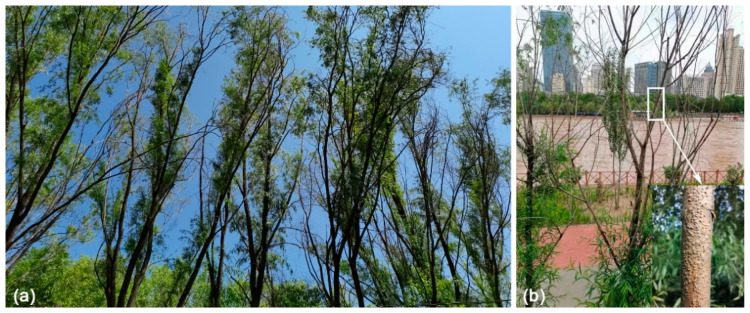
Field sampling in Lanzhou City, Gansu Province: (**a**) death of the willow trees caused by *Cytospora* species; (**b**) symptoms of branches canker.

Willow (*Salix*) trees have been widely cultivated in China because of their strong ecological adaptability, fast growth, easy reproduction, and short rotation period [1]. In terms of horizontal distribution, they are distributed all over the country, with the most concentrated distribution in the northeast, northwest, and southwest regions. These regions have higher elevations or latitudes, and the horizontal distribution of species is extremely rich [2]. However, a large number of willow trees suffer from diseases caused by several fungal pathogens, such as over 20 species of *Cytospora* causing canker disease [3,4] (https://nt.arsgrin.gov/fungaldatabases, accessed on 6 April 2022); over 50 species of *Melampsora* causing rust disease [5,6,7,8,9] (https://nt.ars-grin.gov/fungaldatabases, accessed on 6 April 2022); *Colletotrichum salicis* causing anthracnose disease [10]; and *Rhytisma filamentosum* causing tar-spot disease [11,12]. Among various diseases that affect willow trees, canker and dieback disease caused by *Cytospora* are the main branch and stem diseases in China, especially in the north (Figure 1) [4,13,14,15,16]. The pathogens in *Cytospora* are host-dominant, and usually invade weak trees. There are many factors that cause tree weakness, such as meteorological factors including temperature, humidity, rainfall, sunshine, airflow, etc. Meanwhile, tree age, tree species, slope aspect, soil, nursery management, planting density, and stand tending management technology are also factors that cause Cytospora canker disease. These factors influence each other and work together [17].

Symptoms of Cytospora canker disease associated with willow trees vary according to the stage of disease development. The disease infects the inner bark and causes the sapwood to sink slightly and discolor [18]. Several prominent fruiting bodies form and are buried or semi-buried under the bark, solitary, or in clusters [4]. Under moist conditions, the conidia emerge from the conidiomata in the form of colored and coiled tendrils [4]. Usually, these conidiomata develop in the cankers on bark but may also exist in healthy plant tissue and can be isolated from sound bark, xylem, and leaves of many tree species [19,20]. The genus *Cytospora* (Cytosporaceae, Diaporthales) was established by Ehrenberg [21], which comprises important phytopathogenic, saprobic, and endophytic fungi [4,18]. Species of *Cytospora* inhabit a wide variety of hosts that include economically and ecologically important trees (e.g., Elaeagnaceae, Juglandaceae, Rosaceae, Salicaceae, Ulmaceae) [22,23,24,25,26]. About 150 species of *Cytospora* in total have been discovered on dieback and stem canker in over 130 species of woody hosts [18,27,28,29,30,31,32,33].

Currently, use of multiphase approaches such as morphological and phylogenetic analyses to define species of *Cytospora* has been proposed [4]. In morphology, presence or absence of conceptacle, quantity and arrangement of locule(s), shape and size of conidiophores, and conidial size are significantly taxonomic [3,4]. In phylogeny, the current studies use multigene approaches such as the internal transcribed spacer (ITS), partial actin (*act*), RNA polymerase II subunit (*rpb2*), translation elongation factor 1-α (*tef1-α*), and beta-tubulin (*tub2*) to perform phylogenetic analysis [4,29,32,33].

Over 20 *Cytospora* species infect willow trees all over the world (https://nt.ars-grin.gov/fungaldatabases, accessed on 6 April 2022). An increase in the number of *Cytospora* species isolated from willow cankers has also been reported in China recently. Fan et al. [22] concluded that six species of *Cytospora* from *Salix* were observed in northern China: *C. chrysosperma*, *C. fugax*, *C. leucosperma*, *C. nivea*, *C. populina*, and *C. rostrata*. Wang et al. [3] identified six *Cytospora* species occurring on poplar and willow: *C. atrocirrhata*, *C. chrysosperma*, *C. davidiana*, *C. fugax*, *C. kantschavelii*, and *C. translucens*. The diversity of *Cytospora* on willows deserves further exploration for better disease management. There is an urgent need for systematic studies and re-evaluation to facilitate the identification of *Cytospora* from *Salix* in China. The objectives of this study were (1) to determine species of *Cytospora* from willow trees in China; (2) to confirm the pathogenicity of two new *Cytospora* species; (3) to determine the effects of temperature, pH, and carbon source on the mycelial growth of the new *Cytospora* species; and (4) to evaluate the geographical distribution of *Cytospora* species associated with willow canker disease in China.

## 2. Materials and Methods

### 2.1. Specimens and Strains

Sixty-two specimens were collected from diseased branches or twigs of *Salix matsudana*, *Salix psammophila*, and *Salix cupularis* distributed in China. Sixty-six *Cytospora* strains were isolated by removing a mucoid spore mass from conidiomata and/or ascomata. The suspension was spread over the surface of potato dextrose agar (PDA) (potato, 200 g; glucose, 20 g; agar, 20 g; distilled water, to complete 1000 mL) media, which were incubated at 25 °C in darkness until spores germinated. Hyphal tips were removed to a new PDA plate twice to obtain a pure culture. Specimens were preserved in the Museum of Beijing Forestry University (BJFC). Strains were deposited in the China Forestry Culture Collection Centre (CFCC).

### 2.2. Microscopic Observations

The ascomata and conidiomata formed on the branches and/or twigs of *Salix* were photographed using the Leica stereomicroscope (M205) (Leica Microsystems, Wetzlar, Germany). Over 30 conidiomata (and/or ascomata) and 50 conidia and/or ascospores were selected randomly to measure their lengths and widths using a Nikon Eclipse 80i microscope (Nikon Corporation, Tokyo, Japan) equipped with a Nikon digital sight DS-Ri2 high-definition color camera with differential interference contrast (DIC). Cultural characteristics of strains plated onto PDA and incubated at 25 °C in darkness were recorded after seven and thirty days according to the color charts of Rayner [34].

### 2.3. DNA Extraction, Amplification, and Phylogeny

Total genomic DNA was harvested from axenic cultures on PDA using CTAB method [35]. PCR amplifications and sequencing of ITS, *act*, *rpb2*, *tef1-α*, and *tub2* genes were performed. The PCR conditions and primers are listed in Table S1. PCR products were electrophoresed in 1% agarose gel and the DNA was sequenced by the Tsingke Biotechnology Company Limited. A preliminary identification of strains was based on BLAST using the ITS sequences to confirm the strains obtained in the current study grouped in the genus *Cytospora*. Twenty-two representative strains were selected for the following analyses excluding the strains having parallel ITS sequence, morphological features, host, and location for each species. The sequences generated from this study were supplemented with other sequences obtained from GenBank (Table S2) based on recent publications of *Cytospora* [4,26,32]. For the five individual datasets (ITS, *act*, *rpb2*, *tef1-α*, and *tub2*), sequences alignments were made using MAFFT v. 6.0 [36] and adjusted using MEGA v. 6.0 [37]. Ambiguous regions were excluded from alignments.

Phylogenetic analyses were carried out with maximum parsimony (MP), maximum likelihood (ML), and Bayesian Inference (BI) analyses. MP analysis was computed with PAUP v. 4.0b10 [38] using a heuristic search option of 1000 random-addition sequences with a tree bisection and reconnection (TBR) branch swapping algorithm. ML analysis was computed with GTR+G+I model of site substitution using PhyML v. 3.0 [39] following the instruction of Fan et al. [4]. BI analysis was computed using the best-fit evolutionary models for each partitioned locus estimated in MrModeltest v. 2.3 [40], with a Markov Chain Monte Carlo (MCMC) algorithm in MrBayes v. 3.1.2 [41]. Phylograms were viewed in Figtree v. 1.3.1 [42]. Sequence data were deposited in GenBank. The multilocus sequence alignment and the trees obtained were deposited in TreeBASE (study ID S29245).

### 2.4. Pathogenicity Test

After species identification, healthy cutting plants of *Salix matsudana* inoculated with two novel species (*C. alba* and *C. paracinnamomea*) to determine the relative pathogenicity. For mycelial inoculation, the ex-holotype strains (*C. alba*: CFCC 55462; *C. paracinnamomea*: CFCC 55453) were cultured for a duration of five days at room temperature (25 °C) in darkness. Cuttings of plants were scalded using a 5-mm-diameter sterile inoculation loop, putting a PDA mycelial plug on each burn wound. Moistened cotton was placed directly above each disc, and all were covered with parafilm. Ten inoculation dots were set for each isolate. Ten replicates were inoculated using uncolonized PDA plugs to serve as controls. After a week, the parafilm and cotton wool were removed. The inoculated plants were maintained in the field to observe the symptoms of willow canker and expansion of lesion length. Re-isolation and morphological observation of fungal pathogens were performed to verify Koch’s postulates [43].

### 2.5. Temperature, pH, and Carbon Source Tests

Ex-holotype strains (*C. alba*: CFCC 55462; *C. paracinnamomea*: CFCC 55453) were selected to evaluate the effects of temperature, pH, and carbon source on mycelial growth of *Cytospora*. For the temperature variation test, a 5-mm-diameter mycelial plug was inoculated onto PDA media in triplicate and incubated in the dark at 0–40 °C with a 5 °C gradient. For the pH variation test, the PDA was adjusted with 1 mol/L NaOH and 1 mol/L HCl to obtain pH values ranging from 3.0 to 11.0 at an interval of 1.0. A 5-mm-diameter mycelial plug was inoculated in the medium in triplicate and the cultures were incubated in the dark at 25 °C [44,45]. For the investigation of the utilization of carbon sources, the strains were incubated in triplicate in the dark at 25 °C and the PDA was used as the base medium replacing glucose by the tested carbon sources. The 20 g of glucose was replaced by 20 g of fructose, galactose, maltose, sucrose, and xylose [44,45]. Measurements of colony diameter were made every 24 h for four days. Data were analyzed using IBM SPSS Statistics v. 19.0 by one-way analysis of variance (ANOVA). Linear Regression was used to estimate optimum growth temperature and pH value. Figures were output using Microsoft Excel.

### 2.6. Geographic Distribution

The administrative map of China used in this study includes 34 provincial administrative regions (http://www.tianditu.gov.cn, accessed on 1 March 2022). The dry and wet conditions of China’s land surface followed Wu et al. [46] to divide China into four dry and wet regions, namely, humid, semi-humid, semi-arid, and arid. *Salix* species distribution information was mainly obtained from the Flora of China [47], the Sylva Sinica [48], the National Specimen Information Infrastructure (NSII) (http://www.nsii.org.cn/, accessed on 6 April 2022), and the Atlas of Woody Plants in China [49]. *Cytospora* species distribution information was mainly obtained from the current study (66 strains, 8 species), original records (633 records, 7 species) [13,50,51], and recent publications (63 strains, 11 species) [3,4,16,22]. A total of 13 geographically distributed *Cytospora* species were studied, including eight collected in this study (*C. ailanthicola*, *C. alba*, *C. chrysosperma*, *C. gigaspora*, *C. nivea*, *C. paracinnamomea*, *C. rostrata*, and *C. sophoriopsis*) and five previously reported (*C. atrocirrhata*, *C. fugax*, *C. leucosperma*, *C. populina*, and *C. translucens*). Statistical tests were performed with Poisson regression in R [52].

## 3. Results

### 3.1. Phylogenetic Analyses

**Figure 2:**
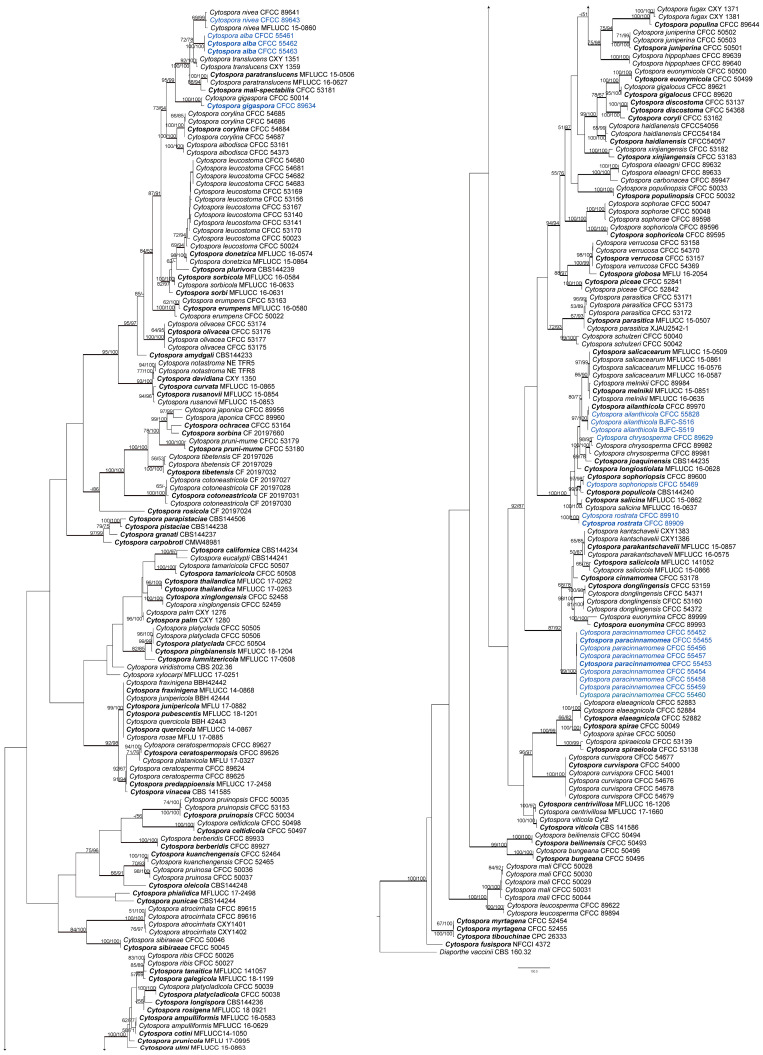
Maximum parsimony phylogram of *Cytospora* based on combined ITS, *act*, *rpb2*, *tef1-α*, and *tub2* genes. MP and ML bootstrap support values above 50% are shown at the first and second position. Thickened branches represent posterior probabilities above 0.95 from BI. Ex-type strains are in bold. Strains from the current study are in blue.

The combined matrix of five genes of *Cytospora* included 3189 characters with gaps (ITS, *act*, *rpb2*, *tef1-α*, and *tub2*, included 604, 351, 726, 843, and 665, respectively). The concatenated alignment comprised sequences from 255 strains and *Diaporthe vaccinii* CBS 160.32 was selected as the outgroup. The alignment contained 189 parsimony-uninformative characters, 1445 characters were variable and parsimony-informative. MP analysis generated 200 equally parsimonious trees with similar clade topologies, one of which was presented in Figure 2 (TL = 9814, CI = 0.305, RI = 0.835, RC = 0.254). In ML analysis, the matrix had 1967 distinct alignment patterns. Estimated base frequencies are as follows: A = 0.244805, C = 0.285887, G = 0.239268, T = 0.230041; substitution rates: AC = 1.258949, AG = 3.497398, AT = 1.467433, CG = 0.961514, CT = 6.191047, GT = 1.000000; gamma distribution shape parameter: α = 0.366687. For BI analysis, the best-fit model of nucleotide evolution was deduced on the AIC (ITS and *act*: GTR+I+G; *rpb2* and *tef1-α*: TrN+I+G; *tub2*: HKY+I+G). ML and Bayesian analyses did not significantly differ from the MP analysis.

The 21 strains obtained in this study clustered in eight clades based on the combined ITS/*act*/*rpb2*/*tef1-α*/*tub2* tree as well as each gene tree except for ITS tree (Figures S1–S5 and Figure 2). The three strains CFCC 55461–55463 grouped as one clade were phylogenetically distinct from all *Cytospora* species with a high statistical support (MP/ML/BI = 100/100/1). Nine strains (CFCC 55452–CFCC 55460) were grouped together (MP/ML/BI = 99/100/1) and distinguished from other *Cytospora* species. These two separate clades are herein described as novel species.

### 3.2. Taxonomy

*Cytospora* species on *Salix* herein include two novel species (*C. alba* and *C. paracinnamomea*) and six known species collected from *Salix* (*C. ailanthicola*, *C. chrysosperma*, *C. gigaspora*, *C. nivea*, *C. rostrata*, and *C. sophoriopsis*). The asexual morph of *C. alba* as well as the sexual and asexual morph of *C. paracinnamomea* were described in this study.

**Figure 3:**
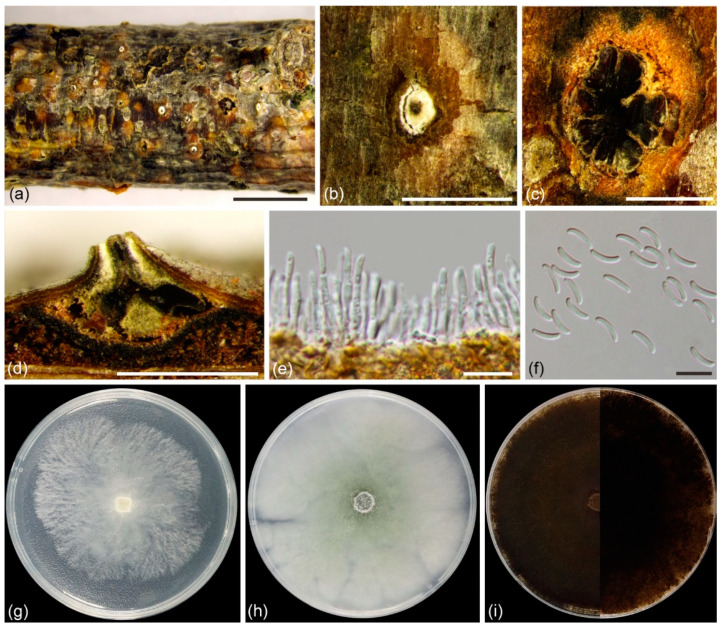
*Cytospora alba* (BJFC CF20201001) from *Salix matsudana*: (**a**,**b**) habit of conidiomata on twig; (**c**) transverse section of conidioma; (**d**) longitudinal section through conidioma; (**e**) conidiophores and conidiogenous cells; (**f**) conidia. Scale bars: 5 mm (**a**); 1 mm (**b**–**d**); 10 µm (**e**,**f**). Cultures on PDA (**g**–**i**, CFCC 55402): (**g**) 2 days; (**h**) 7 days; (**i**) 30 days, front side at left and back side at right.

***Cytospora alba*** L. Lin and X.L. Fan, sp. nov. (Figure 3)

*MycoBank*: MB 841281

*Etymology*: The name reflects the white ectostromatic disc.

*Description*: Asexual morph: Conidiomata labyrinthine cytosporoid, immersed in bark, erumpent when mature, discoid to conical, 990–1930 µm in diam, with multiple locules. Conceptacle present. Ectostromatic disc white to primrose, circular to ovoid, 270–570 µm in diam, with single ostiole per disc in center. Ostiole circular to ovoid, hazel to olivaceous at the same level as disc surface, 93–216 µm in diam. Locules complex with irregular shapes, subdivided by invaginations with common walls. Conidiophores hyaline, unbranched, or occasionally branched at bases, 10–18 × 1.0–2.5 µm (av. = 13.8 ± 1.6 × 1.7 ± 0.2 µm, *n* = 30). Conidiogenous cells enteroblastic, phialidic, subcylindrical to cylindrical, 3.0–6.5 × 1.0–2.0 µm (av. = 5.1 ± 0.9 × 1.5 ± 0.2 µm, *n* = 30). Conidia hyaline, unicellular, eguttulate, elongate-allantoid, 6.0–9.0 × 1.5–2.0 µm (av. = 8.2 ± 0.5 × 1.7 ± 0.1 µm, *n* = 50). Sexual morph: not observed.

*Culture characteristics*: Cultures on PDA initially white, growing to 6.5 cm after 2 days, entirely covering the 9-cm Petri dish after 3 days and becoming greyish yellow green after 7 days. Colonies pale mouse grey and flat with a uniform texture after one month.

*Specimens examined*: CHINA, Gansu Province: Lanzhou City, Yongdeng County, 102°49′27″ E, 36°44′20″ N, from branches of *Salix matsudana*, X.L. Fan, N. Jiang, and C. Peng, 20 October 2020 (holotype BJFC CF20201001, ex-holotype living culture CFCC 55462; ibid., living culture CFCC 55461). Gansu Province: Lanzhou City, Yongdeng County, 102°49′26.7″ E, 36°44′23.2″ N, from branches of *Salix matsudana*, X.L. Fan, N. Jiang, and C. Peng, 20 October 2020 (paratype BJFC CF20201011, ex-paratype living culture CFCC 55463).

*Notes*: *Cytospora alba* is associated with canker disease of *Salix matsudana* in China. It is close to *C. mali-spectabilis* in the phylogenetic diagram (Figure 2). It can be distinguished from *C. mali-spectabilis* by smaller conidia (6.0–9.0 × 1.5–2.0 vs. 9.0–10.0 × 1.5–2 µm in *C. mali-spectabilis*) and the lack of a central column in the conidiomata [26]. *Cytospora alba* has black conceptacle surrounding the asexual stroma, whereas *C. mali-spectabilis* lacks a conceptacle [26]. Furthermore, *C. alba* has multiloculate conidiomata sharing a larger single ostiole (93–216 vs. 60–84 µm) than *C. mali-spectabilis* [26].

***Cytospora ailanthicola*** X.L. Fan and C.M. Tian, Persoonia 45: 13, 2020.

*Descriptions*: See Fan et al. [4].

*Materials examined*: CHINA, Gansu Province: Gannan Autonomous Prefecture, Lintan County, 103°21′20.23″ E, 34°41′33.9″ N, from branches of *Salix matsudana*, 5 August 2012, X.L. Fan (BJFC CF20210502, living culture CFCC 55828). Ningxia Autonomous Region: Zhongwei City, 105°02′17.41″ E, 37°28′43.00′′ N, from branches of *Salix matsudana*, 3 June 2012, X.L. Fan, BJFC-S516. Ningxia Autonomous Region: Yinchuan City, 106°12′28.52″ E, 38°14′07.11″ N, from branches of *Salix matsudana*, 5 June 2012, X.L. Fan, BJFC-S519.

*Notes*: *Cytospora ailanthicola* was introduced by Fan et al. [4] causing canker disease on *Ailanthus altissima* in China. In the current study, we collected specimens of this species associated with twigs and branches of *Salix matsudana*. Although *C. ailanthicola* was closely related to *C. salicacearum* and *C. melnikii* in the phylogram, they can be distinguished by morphological characters. Morphologically, *C. ailanthicola* has multiple locules subdivided frequently by invaginations with common walls, whereas *C. salicacearum* and *C. melnikii* have conidiomata with 1–2 locule(s) and a single locule, respectively [4,22]. In addition, this is the first time that *C. ailanthicola* has been reported from willow tree.

***Cytospora chrysosperma*** (Pers.) Fr., Syst. Mycol. 2: 542, 1823.

Basionym. *Sphaeria chrysosperma* Pers., Neues Mag. Bot. 1: 82, 1794.

Synonym. *Naemaspora chrysosperma* (Pers.) Pers., Observ. Mycol. (Lipsiae) 1: 80, 1796.

=*Valsa sordida* Nitschke, Pyrenomyc. Germ. 2: 203, 1870.

*Descriptions*: See Fan et al. [22].

*Material examined*: CHINA, Shaanxi Province: Yulin City, Airport East Road, 109°39′54.73″ E, 38°19′21.16″ N, from branches of *Salix psammophila*, 1 August 2013, X.L. Fan (BJFC-S975, living culture CFCC 89629).

*Notes*: *Cytospora chrysosperma*, the type species of *Cytospora* [53], is considered a significant plant pathogen causing dieback and canker disease [4,18]. Previous studies have concluded that many species of *Cytospora* have a similar morphology (such as conidiomata cytosporoid and conceptacle absent) as *C. chrysosperma*, which indicated that these species should be regarded as a species complex [4,18]. Recently, Fan et al. [4] revisited ten related *Cytospora* species as belonging to the *C. chrysosperma* complex based on multigene analyses.

***Cytospora gigaspora*** C.M. Tian, X.L. Fan, and K.D. Hyde, Phytotaxa 197: 232, 2015.

*Description*: See Fan et al. [24].

*Material examined*: CHINA, Shaanxi Province: Yulin City, Airport East Road, 109°39′54.73″ E, 38°19′21.16″ N, on twigs and branches of *Salix psammophila*, 1 August 2013, X.L. Fan (BJFC-S975, living culture CFCC 89634).

*Notes*: *Cytospora gigaspora* was originally observed on twigs and branches of *Salix psammophila* in Shaanxi Province [24]. After that, Fan et al. [4] added specimens from Shanxi. *Cytospora gigaspora* differs from *Cytospora nivea* by having flat locules and larger conidia [4].

***Cytospora nivea*** (Hoffm.) Sacc., Michelia 2: 264, 1881.

Basionym. *Sphaeria nivea* Hoffm., Veg. Crypt. 1: 28, 1787.

Synonyms. *Leucostoma niveum* (Hoffm.) Höhn., Mitt. Bot. Inst. Tech. Hochsch. Wien 5: 58, 1928.

=*Valsa nivea* (Hoffm.) Fr., Summa Veg. Scand., Section Post. (Stockholm): 411, 1849.

*Descriptions*: See Fan et al. [4].

*Material examined*: CHINA, Shaanxi Province: Yulin City, Hongshi Gorge, 109°42′00.69″ E, 38°19′32.43″ N, on twigs and branches of *Salix psammophila*, 29 July 2013, X.L. Fan (BJFC-S979, living culture CFCC 89643).

*Notes*: *Cytospora nivea* commonly occurred along with *C. chrysosperma* in association with hosts in the Salicaceae [4,22]. *Cytospora nivea* has a unique characteristic of immersed conidiomata with black conceptacle, multiple locules, and gelatinous conidial tendrils under wet conditions [22]. *Cytospora nivea* have been recorded in Gansu, Ningxia, Shaanxi, and Xinjiang of China [4,13,22,51].

**Figure 4:**
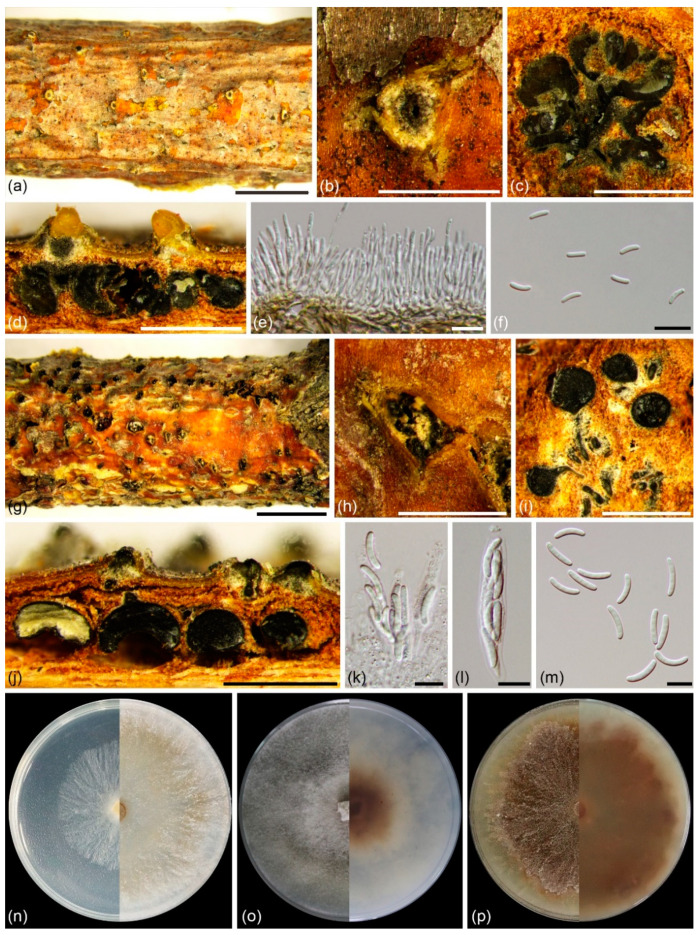
*Cytospora paracinnamomea* (BJFC CF20201003) from *Salix matsudana*: (**a**,**b**) habit of conidiomata on twig; (**c**) transverse section of conidioma; (**d**) longitudinal section through conidiomata; (**e**) conidiophores and conidiogenous cells; (**f**) conidia; (**g**,**h**) habit of ascomata on twig; (**i**) transverse section of ascoma; (**j**) longitudinal section through ascomata; (**k**,**l**) asci; (**m**) ascospores. Scale bars: 5 mm (**a**,**g**); 1 mm (**b**–**d**,**g**,**h**); 10 µm (**e**,**f**,**k**–**m**). Cultures on PDA (**n**–**p**, CFCC 55453): (**n**) 2 days at left and 7 days at right; (**o**) 14 days, front side at left and back side at right; (**p**) 30 days, front side at left and back side at right.

***Cytospora paracinnamomea*** L. Lin and X.L. Fan sp. nov. (Figure 4)

*MycoBank*: MB 841282

*Etymology*: The name reflects the similar culture characteristics with *C. cinnamomea*.

*Description*: Asexual morph: Conidiomata labyrinthine cytosporoid, immersed in bark, erumpent when mature, discoid to conical, 620–2910 µm in diam, with multiple locules. Conceptacle absent. Ectostromatic disc pale luteous to amber, nearly flat, ovoid to ellipsoid, 380–745 µm diam, with single ostiole per disc in center. Ostiole conspicuous, circular to ovoid, umber to black at the same or lower level as disc surface, 235–585 µm diam. Locules numerous, irregular arrangement with individual walls. Conidiophores borne along locules, hyaline, unbranched, or occasionally branched at base, 12.5–20.0(–28.0) × 1.0–1.5 µm (av. = 16.7 ± 3.1 × 1.4 ± 0.1 µm, *n* = 30). Conidiogenous cells enteroblastic, phialidic, subcylindrical to cylindrical, 4.0–8.5 × 1.0–2.0 µm (av. = 6.0 ± 1.1 × 1.4 ± 0.2 µm, *n* = 30). Conidia hyaline, allantoid, smooth, aseptate, thin-walled, (4.0–)5.0–6.2(–6.6) × 1.3–1.7(–2.0) µm (av. = 5.6 ± 0.4 × 1.5 ± 0.1 µm, *n* = 50). Sexual morph: Ascomata euvalsoid, monostichous, immersed in bark, erumpent through bark surface in a large area, scattered, with 4–7 perithecia arranged irregularly. Conceptacle absent. Ectostromatic disc pale luteous, usually surrounded by ostiolar necks, triangular to circular, 530–1020 µm in diam., with 7–17 ostioles arranged irregularly. Ostioles numerous, greenish olivaceous to black when mature, at the same or below level as disc, arranged irregularly in a disc, 50–90 µm in diam. Perithecia primrose to black when mature, flask-shaped to spherical, arranged irregularly, 355–695 µm in diam. Asci hyaline, with refractive, chitinoid ring in nonamyloid apical apparatus, clavate to elongate-obovoid, 40.0–50.0 × 8.0–10.0 (av. = 46.5 ± 3.2 × 9.0 ± 0.4, *n* = 30) µm, 8-spored. Ascospores hyaline, biseriate to multiseriate, elongate to allantoid, thin-walled, aseptate, 12.0–16.0 × 2.5–4.0 µm (av. = 14.2 ± 0.7 × 3.0 ± 0.2 µm, *n* = 50).

*Culture characteristics*: Cultures on PDA initially white, growing up to 9 cm after 2 days, and becoming umber after 14 days in center. Colonies thick, concentric circles with a uniform texture, lacking aerial mycelium.

*Specimens examined*: CHINA, Gansu Province: Lanzhou City, Yongdeng County, 102°49′28″ E, 36°44′21″ N, from branches of *Salix matsudana*, X.L. Fan and L. Lin, 20 October 2020 (holotype BJFC CF20201003, ex-holotype living culture CFCC 55453; ibid., living culture CFCC 55452, CFCC 55454). Gansu Province: Lanzhou City, Yongdeng County, 102°49′26.7″ E, 36°44′22.4″ N, from branches of *Salix matsudana*, X.L. Fan and L. Lin, 20 October 2020 (paratype BJFC CF20201002, ex-paratype living culture CFCC 55455; ibid., living culture CFCC 55456–55460).

*Notes*: *Cytospora paracinnamomea* is associated with canker disease of *Salix matsudana* in China. It can be distinguished from its phylogenetically closely related species *C. cinnamomea* (ex-holotype isolate CFCC 53178) and *C. donglingensis* (ex-type isolate CFCC 53159). Morphologically, it differs from *C. cinnamomea* by larger ostiole (235–585 vs. 50–63 µm) [26]. It differs from *C. donglingensis* by cultures on PDA becoming umber after 14 days in the center while cultures of *C. donglingensis* on PDA become straw after 10 days. Phylogenetically, the current nine strains (CFCC 55452–CFCC 55460) grouped in a separate clade with high statistical support (MP/ML/BI = 99/100/1).

***Cytospora rostrata*** C.M. Tian and X.L. Fan, Mycotaxon 129: 307, 2014.

*Descriptions*: See Fan et al. [22]

*Materials examined*: CHINA, Gansu Province: Gannan Autonomous Prefecture, Diebu County, 103°23′34.20″ E, 34°04′48.85″ N, from stems of *Salix cupularis*, 9 August 2012, Y.M. Liang and X.L. Fan (BJFC S726, living culture CFCC 89909); Gansu Province: Ganan City, Diebu County, 103°23′36.60″ E, 34°04′48.35″ N, from stems of *Salix cupularis*, 9 August 2012, Y.M. Liang and X.L. Fan (BJFC S727, living culture CFCC 89910).

*Notes*: *Cytospora rostrata* can be distinguished from other species by having thornlike ostiolar beak [22]. This species has only been found in *Salix cupularis* in Gansu Province.

***Cytospora sophoriopsis*** X.L. Fan and C.M. Tian, Persoonia 45: 39, 2020.

*Descriptions*: See Fan et al. [4]

*Material examined*: CHINA, Gansu Province: Lanzhou City, Yongdeng County, 102°49′29″ E, 36°44′22″ N, from branches of *Salix matsudana*, X.L. Fan, N. Jiang, and C. Peng, 20 October 2020 (BJFC CF20201005, living culture CFCC 55469).

*Notes*: *Cytospora sophoriopsis* has scattered conidiomata without conceptacles, numerous locules subdivided by invaginations with common wall, and conidia measuring 4.0–4.5 × 1.0–1.5 µm [4]. Phylogenetically, the isolate in the current study (CFCC 55469) formed a fully supported clade with sequences from the ex-type isolate of *C. sophoriopsis*. In addition, the current study extends the host range of *C. sophoriopsis* to *Salix matsudana* in China.

### 3.3. Pathogenicity Test

**Figure 5:**
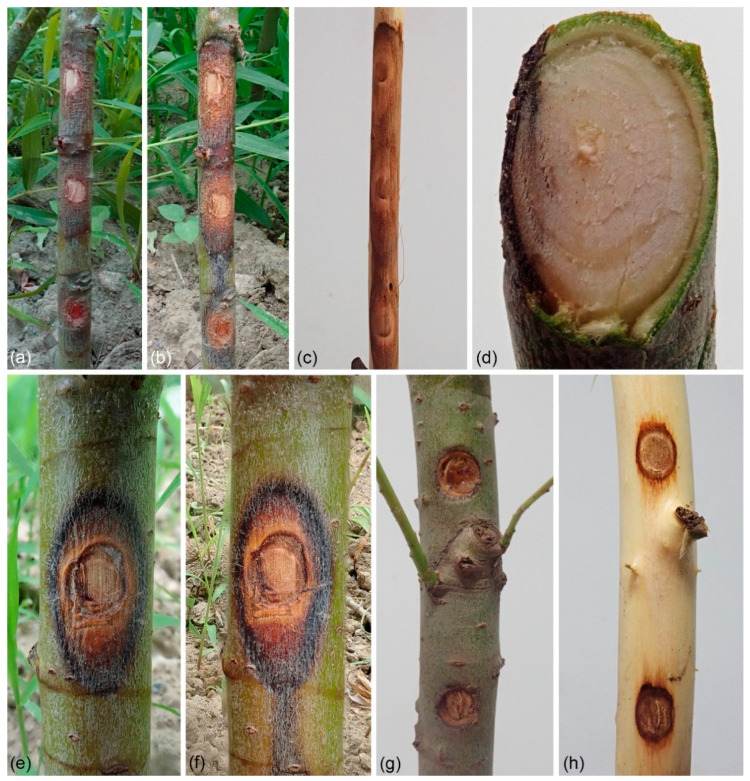
Stem blight symptoms on *Salix matsudana* caused by *Cytospora* species: (**a**–**d**) stem blight symptoms on *Salix matsudana* caused by *Cytospora alba* (CFCC 55462). Symptoms after (**a**) one week and (**b**) two weeks. (**c**) Resinous canker formed after inoculation with *C. alba*. (**d**) Longitudinal section through pathogenic site. (**e**,**f**) Stem blight symptoms on *Salix matsudana* caused by *Cytospora paracinnamomea* (CFCC 55453). Symptoms after (**e**) one week and (**f**) two weeks. (**g**,**h**) Blank control group, no symptoms on *Salix matsudana* twigs after three weeks of inoculation with agar block.

Through pathogenicity tests, both *C. alba* (CFCC 55462) and *C. paracinnamomea* (CFCC 55453) were confirmed as pathogens on the willow stems (Figure 5). Sunken and resinous cankers were obvious on the stems when the parafilm was removed after 7 days. The enlarged cankers lead further to wilting and consequent death of the plants. No symptoms were observed in the non-inoculated controls. *Cytospora alba* (CFCC 55462) was found to be more virulent than *C. paracinnamomea* (CFCC 55453) with the lesion presence frequency reaching 100% and canker length averaging 53 mm after 7 days. The average lesion caused by *C. paracinnamomea* (CFCC 55453) was 36 mm in length after 7 days. All *Cytospora* species were reisolated from the lesions or reproductive structures on inoculated plants.

### 3.4. Temperature, pH, and Carbon Source Tests

The effects of temperature, pH, and carbon source tests were performed with the ex-holotype strains (*C. alba*: CFCC 55461 and *C. paracinnamomea*: CFCC 55453). The regression equations and the estimated optimum growth temperature and pH value are presented in Table S3.

**Figure 6:**
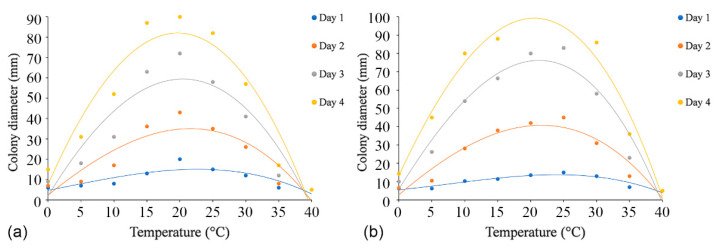
Effects of temperature on the growth of *Cytospora* species. (**a**) *Cytospora alba* (CFCC 55462): y_Day1_ = −0.0005x^3^ + 0.0035x^2^ + 0.6438x + 4.6768 (R^2^ = 0.7348); y_Day2_ = −0.0009x^3^ − 0.033x^2^ + 2.6289x + 2.3212 (R^2^ = 0.8171); y_Day3_ = −0.0007x^3^ − 0.1103x^2^ + 5.3437x + 1.9394 (R^2^ = 0.8513); y_Day4_ = −0.0004x^3^ − 0.1762x^2^ + 7.4157x + 7.4949 (R^2^ = 0.9055). (**b**) *Cytospora paracinnamomea* (CFCC 55453): y_Day1_ = −0.0006x^3^ + 0.0147x^2^ + 0.3385x + 5.5222 (R^2^ = 0.8937); y_Day2_ = −0.0009x^3^ − 0.0406x^2^ + 3.0735x + 2.7606 (R^2^ = 0.9161); y_Day3_ = −0.0017x^3^ − 0.0843x^2^ + 5.8622x + 5.7758 (R^2^ = 0.944); y_Day4_ = −0.0014x^3^ − 0.1473x^2^ + 7.7947x + 13.211 (R^2^ = 0.9641).

Colonies of both *C. alba* (CFCC 55461) and *C. paracinnamomea* (CFCC 55453) grew in the temperature range of 0–30 °C, but not at 35 °C or 40 °C. The fastest mycelial growth of *C. alba* (CFCC 55461) occurred at 20 °C, and *C. paracinnamomea* (CFCC 55453) occurred at 20 °C or 25 °C, with colonies of both reaching a diameter of 90 mm after four days. The least mycelial growth of *C. alba* (CFCC 55461) and *C. paracinnamomea* (CFCC 55453) all occurred at 0 °C, reaching only 15 mm and 14 mm, respectively, after four days. Based on the regression analysis, the optimal growth after incubation was estimated to occur at 21.1 °C for *C. alba* (CFCC 55461) and at 21.9 °C for *C. paracinnamomea* (CFCC 55453) (Figure 6).

**Figure 7:**
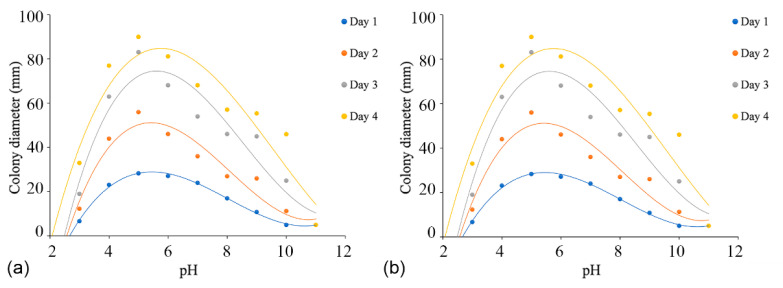
Effects of pH on the growth of *Cytospora* species. (**a**) *Cytospora alba* (CFCC 55462): y_Day1_ = 0.3546x^3^ − 8.5492x^2^ + 61.603x − 110.27 (R^2^ = 0.9957); y_Day2_ = 0.5799x^3^ − 14.068x^2^ + 101.43x − 177.67 (R^2^ = 0.9252); y_Day3_ = 0.6425x^3^ − 16.478x^2^ + 124.19x − 217.02 (R^2^ = 0.8945); y_Day4_ = 0.4139x^3^ − 11.886x^2^ + 95.703x − 151.21 (R^2^ = 0.8693). (**b**) *Cytospora paracinnamomea* (CFCC 55453): y_Day1_ = 0.2383x^3^ − 5.3296x^2^ + 35.599x − 57.712 (R^2^ = 0.9028); y_Day2_ = 0.9923x^3^ − 23.044x^2^ + 160.21x − 287.09 (R^2^ = 0.9761); y_Day3_ = 1.0142x^3^ − 25.35x^2^ + 188.44x − 350.98 (R^2^ = 0.9806); y_Day4_ = 1.037x^3^ − 27.082x^2^ + 208.41x − 391.11 (R^2^ = 0.9361).

Colonies of both *C. alba* (CFCC 55461) and *C. paracinnamomea* (CFCC 55453) grew on PDA in the pH range of 3.0–10.0, but not at a pH of 11.0. For *C. alba* (CFCC 55461), mycelium grew most rapidly at pH 5.0, reaching 90 mm after four days, followed by pH 4.0 and 6.0. Mycelium grew slowly at pH values of 10.0 and 11.0, attaining colony diameters of no more than 30 mm after four days. For *C. paracinnamomea* (CFCC 55453), mycelium grew most rapidly at pH 6.0, reaching 90 mm after three days, followed by pH 5.0 and 7.0. Mycelium grew slowly at pH values of 10.0, attaining colony diameters of no more than 14 mm after four days. Based on the regression analysis, the optimal growth after incubation was estimated to occur at pH 5.6 for *C. alba* (CFCC 55461) and at pH 5.4 for *C. paracinnamomea* (CFCC 55453) (Figure 7).

**Figure 8:**
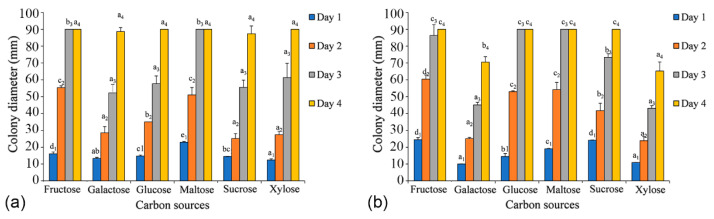
Effects of carbon sources on the growth of *Cytospora* species. (**a**) *Cytospora alba* (CFCC 55462). (**b**) *Cytospora paracinnamomea* (CFCC 55453). A *p*-value < 0.05 was considered significant. Graphs with SE-bars showed the difference in mycelium growth under different conditions.

Both *C. alba* (CFCC 55461) and *C. paracinnamomea* (CFCC 55453) have the ability to grow on all six carbon sources tested. After two days, the growth of mycelia of *C. alba* (CFCC 55461) on the fructose and maltose media was apparently faster than that on other media (*p*-value = 0.0001), and the growth of mycelia of *C. paracinnamomea* (CFCC 55453) on the fructose, glucose, and maltose media was apparently faster than that on other media after two days (*p*-value = 0.0001). The colonies grown on other carbon sources were not significantly different from one another. After three days, mycelium growing on fructose and maltose were the first cultures to reach 90 mm in diameter. For *C. alba* (CFCC 55461), the utilization of sucrose was apparently lower than that of the others, reaching no more than 87 mm growth in diameter after four days. For *C. paracinnamomea* (CFCC 55453), the utilization of xylose was apparently lower than that of the others, reaching no more than 65 mm growth in diameter after four days (Figure 8).

### 3.5. Geographic Distribution

**Figure 9:**
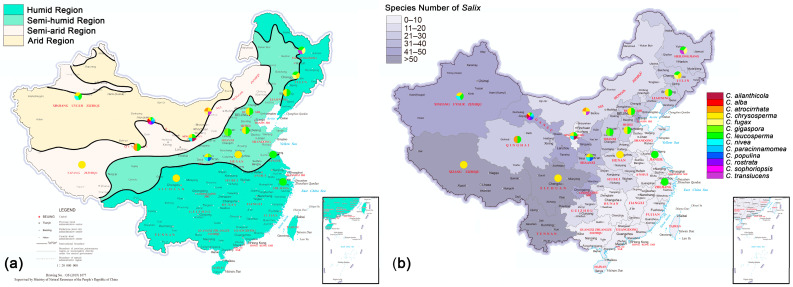
Geographical distribution of *Cytospora* species associated with willow canker disease in China: (**a**) drought conditions and *Cytospora* species richness of each province; (**b**) *Salix* species richness and *Cytospora* species richness of each province.

The geographical distribution of *Cytospora* species from *Salix* shows that *C. chrysosperma* and *C. leucosperma* are the main species observed on *Salix*, followed by *C. nivea* (Figure 9). *Cytospora chrysosperma* and *C. leucosperma* can be found almost in the entire distribution area of *Salix* (12 provinces); *C. nivea* and *C. populina* are mainly distributed in the Yellow River Basin (four and two provinces, respectively). Gansu province has the highest species diversity of *Cytospora* (nine species). The results show that the number of *Cytospora* species from *Salix* in China is more abundant in the northwest and less in the southeast (Figure 9). Poisson regression analysis showed that *Cytospora* species richness was significantly positively correlated with dry and wet areas (*p*-value = 0.024 < 0.05, Estimate = 0.33296). The more drought conditions there were, the more *Cytospora* species richness was recorded. There was no correlation with host *Salix* species richness (*p*-value = 0.303).

## 4. Discussion

As an afforestation tree species with important economic and ecological value, *Salix* can be infected by a variety of fungi [4,6,10,12]. *Cytospora* is a genus in Diaporthales that includes many plant pathogens and endophytes [4,18,27,28,29,54,55]. In the current study, a total of eight *Cytospora* species from *Salix* in China were re-evaluated, including *C. ailanthicola*, *C. alba*, *C. chrysosperma*, *C. gigaspora*, *C. nivea*, *C. paracinnamomea*, *C. rostrata*, *C. sophoriopsis*, and *C. translucens* [3,4,13,16,50,51,56,57]. Among these, *C. alba* and *C. paracinnamomea* were described as novel species in this study. *Cytospora ailanthicola* and *C. sophoriopsis* represent the first record from willow trees in China.

In previous studies, five species (*C. atrocirrhata*, *C. fugax*, *C. leucosperma*, *C. populina*, and *C. translucens*) associated with willow canker disease have also been reported in China [3,4,13,24,51,57]. *Cytospora atrocirrhata* was first reported on branches of willow in Georgia [58], which was described as erumpent conidiomata with distinct conceptacles and single locules and ostiole [24]. Subsequently, this species was reported on poplar and walnut in Inner Mongolia and Qinghai [3,24]. Spielman [53] re-described the sexual and asexual morph of *C. fugax*, which provided important information for the accurate identification of this species. In China, *Cytospora fugax* was previously recorded in Heilongjiang, Inner Mongolia, and Jilin [3]. *Cytospora leucosperma* was recorded in Beijing, Gansu, Hebei, Heilongjiang, Jiangsu, Jilin, Liaoning, Ningxia, Qinghai, Shanxi, Xinjiang, and Zhejiang Provinces in China [4,13,51,57]. *Cytospora populina* was redescribed by Fan et al. [24] on branches of *Salix psammophila* in China. It can be distinguished from other *Cytospora* species by its asci with four ascospores [24]. *Cytospora translucens* was previously discovered on willow and poplar in Beijing, Heilongjiang, Inner Mongolia, and Jilin [3]. Vu et al. [59] added their DNA data; however, they still require a modern illustration and description.

*Cytospora atrocirrhata*, *C. chrysosperma*, *C. fugax*, *C. leucosperma*, *C. nivea*, and *C. translucens* were also reported to infect *Salix* spp. in other countries, e.g., Canada, England, Iran, New Zealand, Poland (https://nt.ars-grin.gov/fungaldatabases/index.cfm, accessed on 6 April 2022). Among these species, *C. chrysosperma* can infect over 110 host species, which is a prominent species of *Cytospora* in the world [4,18]. *Cytospora ailanthicola*, *C. gigaspora*, *C. rostrata*, *C. sophoriopsis* have only been found in China, of which *C. rostrata* has been only found in *Salix cupularis* [3,4,22,23]. Although *C. alba* and *C. paracinnamomea* have been confirmed as pathogens of willow canker disease, they have only been found in Gansu province. Whether they are distributed elsewhere and infect local willow trees are unknown.

The results of pathogenicity tests showed that *C. alba* and *C. paracinnamomea* were pathogens to *Salix matsudana*, which had a high degree of damage. The optimal growth was estimated to occur at 21.1 °C for *C. alba* (CFCC 55461) and at 21.9 °C for *C. paracinnamomea* (CFCC 55453); and at pH 5.6 for *C. alba* (CFCC 55461) and at pH 5.4 for *C. paracinnamomea* (CFCC 55453). Fructose and maltose were utilized better than other tested carbon sources. Based on the biological characterization of these two novel *Cytospora* species, more attention is needed to prevent the occurrence of Cytospora canker disease in the region with an average monthly temperature of approximately 20 °C, such as April, May, September, and October in Southwest, Northwest, and North China. This indicates that both spring and autumn may be a time of high disease risk. Therefore, the understanding of biological characteristics of these *Cytospora* species can be used for distribution prediction. Preventive measures can be taken in advance.

The geographical distribution analysis showed that *Cytospora* on *Salix* was less commonly known in the humid regions than in the arid region, semiarid region, and semi-humid region. Previous studies showed that drought-stressed plants are more prone to Cytospora cankers [60,61]. However, Kristen et al. [62] demonstrated that reduced water availability and a 2–3 °C increase in temperature did not significantly increase the incidence or severity of cankers in inoculated willow plants. Therefore, willows that occur in dry areas are more likely to be infected by *Cytospora* because the trees are weakened by drought stress and poor soil conditions.

## Data Availability

Alignments generated during the current study are available in Tree-BASE (accession http://purl.org/phylo/treebase/phylows/study/TB2:S29245, accessed on 6 April 2022). All sequence data are available in NCBI GenBank following the accession numbers in the manuscript.

## Supplementary Materials

The following supporting information can be downloaded at: https://www.mdpi.com/article/10.3390/jof8040377/s1, Refs. [63,64,65,66,67], Figure S1: Phylogenetic tree of *Cytospora* species based on ML analyses of the ITS gene sequences; Figure S2: Phylogenetic tree of *Cytospora* species based on ML analyses of the *act* gene sequences; Figure S3: Phylogenetic tree of *Cytospora* species based on ML analyses of the *rpb2* gene sequences; Figure S4: Phylogenetic tree of *Cytospora* species based on ML analyses of the *tef1-α* gene sequences; Figure S5: Phylogenetic tree of *Cytospora* species based on ML analyses of the *tub2* gene sequences; Table S1: Genes used in this study with PCR primers, primer DNA sequence, optimal annealing temperature; Table S2: Strains of *Cytospora* used in the molecular analyses in this study; Table S3: The regression equations and the estimated optimum growth temperature, pH value, and carbon source.
