# Supplementary material for: Fungal Richness of Cytospora Species Associated with Willow Canker Disease in China
Source: J Fungi (Basel). 2022 Apr 7;8(4):377. doi: 10.3390/jof8040377 (PMC9030772; doi:10.3390/jof8040377)
Supplement: Supplementary file 1 [file jof-08-00377-s001.zip › Tables S1-S3.pdf]

Table S1. Genes used in this study with PCR primers, primer DNA sequence, optimal annealing temperature.

Locus	Definition	Primers	Primer DNA sequence (5′–3′)	Optimal annealing temp (°C)	References of primers used
ITS	internal transcribed spacer of ribosomal RNA	ITS1	TCCGTAGGTGAACCTGCGG	51	White et al. 1990
		ITS4	TCCTCCGCTTTTGATATGC		
act	actin	ACT-512F	ATGTGCAAGGCCGGTTTCGC	61	Carbone and Kohn, 1999
		ACT-783R	TACGAGTCCTTCTGGCCCAT		
rpb2	RNA polymerase II second largest subunit	RPB2-5F	GA(T/C)GA(T/C)(A/C)G(A/T)GATCA(T/C)TT(T/C)GG	52	Liu et al. 1999
		RPB2-7cR	CCCAT(A/G)GCTTG(T/C)TT(A/G)CCCAT		
tef-1α	translation elongation factor 1-alpha	EF1-688F	CGGTCACTTGATCTACAAGTGC	55	Alves et al. 2008
		EF1-1251R	CCTCGAACTCACCAGTACCG		
tub2	beta-tubulin	Bt2a	GGTAACCAAATCGGTGCTGCTTTG	55	Glass and Donaldson, 1995
		Bt2b	ACCCTCAGTGTAGTGACCCTTGGC		

Alves, A., Crous, P. W., Correia, A., Phillips, A. J. L. 2008. Morphological and molecular data reveal cryptic speciation in Lasiodiplodia theobromae. Fungal Diversity 28:1–13.

Carbone, I., Kohn, L. 1999. A method for designing primer sets for speciation studies in filamentous ascomycetes. Mycologia 91:553–556.

Glass, N. L., Donaldson, G. C. 1995. Development of primer sets designed for use with the PCR to amplify conserved genes from filamentous ascomycetes. Applied and environmental microbiology 61:1323–1330.

Liu, Y. L. Whelen, S., Hall, B. D. 1999. Phylogenetic relationships among ascomycetes: evidence from an RNA polymerase II subunit. Molecular Biology and Evolution 16:1799–1808.

White, T. J., Bruns, T., Lee, S., Taylor, J. 1990. Amplification and direct sequencing of fungal ribosomal RNA genes for phylogenetics. PCR Protocols: A Guide to Methods and Applications 18:315–322.

Table S2. Strains of *Cytospora* used in the molecular analyses in this study.

Species	Strain[1]	Host	Origin	GenBank accession numbers				
				ITS	*act*	*rpb2*	*tef1-α*	*tub2*
Cytospora ailanthicola	**CFCC 55828**	***Salix matsudana***	**Gansu, China**	**OK156410**	**OK303491**	**OK303564**	**OK303627**	**OK303694**
Cytospora ailanthicola	**BJFC-S516**	***Salix matsudana***	**Ningxia, China**	**OK147827**	**OK303492**	**NA**	**NA**	**OK303695**
Cytospora ailanthicola	**BJFC-S519**	***Salix matsudana***	**Ningxia, China**	**OK147829**	**OK303494**	**NA**	**OK303628**	**OK303697**
Cytospora ailanthicola	CFCC 89970	*Ailanthus altissima*	Ningxia, China	MH933618	MH933526	MH933592	MH933494	MH933565
Cytospora albodisca	CFCC 53161	*Platycladus orientalis*	Beijing, China	MW418406	MW422899	MW422909	MW422921	MW422933
Cytospora albodisca	CFCC 54373	*Platycladus orientalis*	Beijing, China	MW418407	MW422900	MW422910	MW422922	MW422934
Cytospora alba	**CFCC 55461**	***Salix matsudana***	**Gansu, China**	**MZ702592**	**OK303454**	**OK303513**	**OK303574**	**OK303641**
Cytospora alba	**CFCC 55462[T]**	***Salix matsudana***	**Gansu, China**	**MZ702593**	**OK303457**	**OK303516**	**OK303577**	**OK303644**
Cytospora alba	**CFCC 55463[T]**	***Salix matsudana***	**Gansu, China**	**MZ702594**	**OK303458**	**OK303517**	**OK303578**	**OK303645**
Cytospora ampulliformis	MFLUCC 16-0583[T]	*Sorbus intermedia*	Russia	KY417726	KY417692	KY417794	NA	NA
Cytospora ampulliformis	MFLUCC 16-0629	*Acer platanoides*	Russia	KY417727	KY417693	KY417795	NA	NA
Cytospora amygdali	CBS 144233[T]	*Prunus dulcis*	California, USA	MG971853	MG972002	NA	MG971659	MG971718
Cytospora atrocirrhata	CFCC 89615	*Juglans regia*	Qinghai, China	KR045618	KF498673	KU710946	KP310858	KR045659
Cytospora atrocirrhata	CFCC 89616	*Juglans regia*	Qinghai, China	KR045619	KF498674	KU710947	KP310859	KR045660
Cytospora atrocirrhata	CXY 1401	*Populus* sp.	Inner Mongolia, China	JX534242	NA	NA	NA	KM034904
Cytospora atrocirrhata	CXY 1402	*Populus* sp.	Inner Mongolia, China	JX534243	NA	NA	NA	KM034903
Cytospora beilinensis	CFCC 50493[T]	*Pinus armandii*	Beijing, China	MH933619	MH933527	NA	MH933495	MH933561
Cytospora beilinensis	CFCC 50494	*Pinus armandii*	Beijing, China	MH933620	MH933528	NA	MH933496	MH933562
Cytospora berberidis	CFCC 89927[T]	*Berberis dasystachya*	Qinghai, China	KR045620	KU710990	KU710948	KU710913	KR045661
Cytospora berberidis	CFCC 89933	*Berberis dasystachya*	Qinghai, China	KR045621	KU710991	KU710949	KU710914	KR045662

Cytospora bungeana	CFCC 50495^{T}	*Pinus bungeana*	Shanxi, China	MH933621	MH933529	MH933593	MH933497	MH933563
Cytospora bungeana	CFCC 50496	*Pinus bungeana*	Shanxi, China	MH933622	MH933530	MH933594	MH933498	MH933564
Cytospora californica	CBS 144234^{T}	*Juglans regia*	California, USA	MG971935	MG972083	NA	MG971645	NA
Cytospora carbonacea	CFCC 89947	*Ulmus pumila*	Qinghai, China	KR045622	KP310842	KU710950	KP310855	KP310825
Cytospora carpobroti	CMW 48981^{T}	*Carpobrotus edulis*	South Africa	MH382812	NA	NA	MH411212	MH411207
Cytospora celtidicola	CFCC 50497^{T}	*Celtis sinensis*	Anhui, China	MH933623	MH933531	MH933595	MH933499	MH933566
Cytospora celtidicola	CFCC 50498	*Celtis sinensis*	Anhui, China	MH933624	MH933532	MH933596	MH933500	MH933567
Cytospora centrivillosa	MFLUCC 16-1206^{T}	*Sorbus domestica*	Italy	MF190122	NA	MF377600	NA	NA
Cytospora centrivillosa	MFLUCC 17-1660	*Sorbus domestica*	Italy	MF190123	NA	MF377601	NA	NA
Cytospora ceratosperma	CFCC 89624	*Juglans regia*	Gansu, China	KR045645	NA	KU710976	KP310860	KR045686
Cytospora ceratosperma	CFCC 89625	*Juglans regia*	Gansu, China	KR045646	NA	KU710977	KP31086	KR045687
Cytospora ceratospermopsis	CFCC 89626^{T}	*Juglans regia*	Shaanxi, China	KR045647	KU711011	KU710978	KU710934	KR045688
Cytospora ceratospermopsis	CFCC 89627	*Juglans regia*	Shaanxi, China	KR045648	KU711012	KU710979	KU710935	KR045689
Cytospora chrysosperma	**CFCC 89629**	***Salix psammophila***	**Shaanxi, China**	**KF765673**	**NA**	**KF765705**	**NA**	**NA**
Cytospora chrysosperma	CFCC 89981	*Populus alba* subsp. *pyramidalis*	Gansu, China	MH933625	MH933533	MH933597	MH933501	MH933568
Cytospora chrysosperma	CFCC 89982	*Ulmus pumila*	Tibet, China	KP281261	KP310835	NA	KP310848	KP310818
Cytospora cinnamomea	CFCC 53178^{T}	*Prunus armeniaca*	Xinjiang, China	MK673054	MK673024	NA	NA	MK672970
Cytospora coryli	CFCC 53162^{T}	*Corylus mandshurica*	Beijing, China	MN854450	NA	MN850751	MN850758	MN861120
Cytospora corylina	CFCC 54684^{T}	*Corylus heterophylla*	Beijing, China	MW839861	MW815951	MW815937	MW815886	MW883969
Cytospora corylina	CFCC 54685	*Corylus heterophylla*	Beijing, China	MW839862	MW815952	MW815938	MW815887	MW883970
Cytospora corylina	CFCC 54686	*Corylus heterophylla*	Beijing, China	MW839863	MW815953	MW815939	MW815888	MW883971
Cytospora corylina	CFCC 54687	*Corylus heterophylla*	Beijing, China	MW839864	MW815954	MW815940	MW815889	MW883972
Cytospora cotini	MFLUCC 14-1050^{T}	*Cotinus coggygria*	Russia	KX430142	NA	KX430144	NA	NA

Cytospora cotoneastricola	CF 20197027	*Cotoneaster* sp.	Tibet, China	MK673072	MK673042	MK673012	MK672958	MK672988
Cytospora cotoneastricola	CF 20197028	*Cotoneaster* sp.	Tibet, China	MK673073	MK673043	MK673013	MK672959	MK672989
Cytospora cotoneastricola	CF 20197030	*Cotoneaster* sp.	Tibet, China	MK673074	MK673044	MK673014	MK672960	MK672990
Cytospora cotoneastricola	CF 20197031[T]	*Cotoneaster* sp.	Tibet, China	MK673075	MK673045	MK673015	MK672961	MK672991
Cytospora curvata	MFLUCC 15-0865[T]	*Salix alba*	Russia	KY417728	KY417694	KY417796	NA	NA
Cytospora curvispora	CFCC 54000[T]	*Corylus heterophylla*	Beijing, China	MW839851	MW815931	MW815945	MW815880	MW883963
Cytospora curvispora	CFCC 54001	*Corylus heterophylla*	Beijing, China	MW839853	MW815932	MW815946	MW815881	MW883964
Cytospora curvispora	CFCC 54676	*Corylus heterophylla*	Beijing, China	MW839854	MW815933	MW815947	MW815882	MW883965
Cytospora curvispora	CFCC 54677	*Corylus heterophylla*	Beijing, China	MW839855	MW815934	MW815948	MW815883	MW883966
Cytospora curvispora	CFCC 54678	*Corylus heterophylla*	Beijing, China	MW839856	MW815935	MW815949	MW815884	MW883967
Cytospora curvispora	CFCC 54679	*Corylus heterophylla*	Beijing, China	MW839857	MW815936	MW815950	MW815885	MW883968
Cytospora davidiana	CXY 1350[T]	*Populus davidiana*	Inner Mongolia, China	KM034870	NA	NA	NA	NA
Cytospora discotoma	CFCC 53137[T]	*Platycladus orientalis*	Beijing, China	MW418404	MW422897	MW422907	MW422919	MW422931
Cytospora discotoma	CFCC 54368	*Platycladus orientalis*	Beijing, China	MW418405	MW422898	MW422908	MW422920	MW422932
Cytospora donetzica	MFLUCC 15-0864	*Crataegus monogyna*	Russia	KY417729	KY417695	KY417797	NA	NA
Cytospora donetzica	MFLUCC 16-0574[T]	*Crataegus monogyna*	Russia	KY417731	KY417697	KY417799	NA	NA
Cytospora donglingensis	CFCC 53159[T]	*Platycladus orientalis*	Beijing, China	MW418412	MW422903	MW422915	MW422927	MW422939
Cytospora donglingensis	CFCC 53160	*Platycladus orientalis*	Beijing, China	MW418414	MW422905	MW422917	MW422929	MW422941
Cytospora donglingensis	CFCC 54371	*Platycladus orientalis*	Beijing, China	MW418413	MW422904	MW422916	MW422928	MW422940
Cytospora donglingensis	CFCC 54372	*Platycladus orientalis*	Beijing, China	MW418415	MW422906	MW422918	MW422930	MW422942
Cytospora elaeagni	CFCC 89632	*Elaeagnus angustifolia*	Ningxia, China	KR045626	KU710995	KU710955	KU710918	KR045667
Cytospora elaeagni	CFCC 89633	*Elaeagnus angustifolia*	Ningxia, China	KF765677	KU710996	KU710956	KU710919	KR045668
Cytospora elaeagnicola	CFCC 52882[T]	*Elaeagnus angustifolia*	China	MK732341	MK732344	MK732347	NA	NA

Cytospora elaeagnicola	CFCC 52883	*Elaeagnus angustifolia*	China	MK732342	MK732345	MK732348	NA	NA
Cytospora elaeagnicola	CFCC 52884	*Elaeagnus angustifolia*	China	MK732343	MK732346	MK732349	NA	NA
Cytospora erumpens	CFCC 50022	*Prunus padus*	Shanxi, China	MH933627	MH933534	NA	MH933502	MH933569
Cytospora erumpens	MFLUCC 16-0580[T]	*Salix × fragilis*	Russia	KY417733	KY417699	KY417801	NA	NA
Cytospora erumpens	CFCC 53163	*Prunus padus*	Xinjiang, China	MK673059	MK673029	MK673000	MK672948	MK672975
Cytospora eucalypti	CBS 144241	*Eucalyptus globulus*	California, USA	MG971907	MG972056	NA	MG971617	MG971772
Cytospora euonymicola	CFCC 50499[T]	*Euonymus kiautschovicus*	Shaanxi, China	MH933628	MH933535	MH933598	MH933503	MH933570
Cytospora euonymicola	CFCC 50500	*Euonymus kiautschovicus*	Shaanxi, China	MH933629	MH933536	MH933599	MH933504	MH933571
Cytospora euonymina	CFCC 89993[T]	*Euonymus kiautschovicus*	Shanxi, China	MH933630	MH933537	MH933600	MH933505	MH933590
Cytospora euonymina	CFCC 89999	*Euonymus kiautschovicus*	Shanxi, China	MH933631	MH933538	MH933601	MH933506	MH933591
Cytospora fraxinigena	BBH 42442	*Fraxinus ornus*	NA	MF190133	NA	NA	NA	NA
Cytospora fraxinigena	MFLUCC 14-0868[T]	*Fraxinus ornus*	Italy	MF190133	NA	NA	NA	NA
Cytospora fugax	CXY 1371	*Populus simonii*	Jilin, China	KM034852	NA	NA	NA	KM034891
Cytospora fugax	CXY 1381	*Populus ussuriensis*	Heilongjiang, China	KM034853	NA	NA	NA	KM034890
Cytospora fusispora	NFCCI 4372	NA	India	MN227694	NA	NA	NA	NA
Cytospora galegicola	MFLUCC 18-1199[T]	*Galega officinalis*	Forlì-Cesena, Italy	MK912128	MN685810	MN685820	NA	NA
Cytospora gigalocus	CFCC 89620[T]	*Juglans regia*	Qinghai, China	KR045628	KU710997	KU710957	KU710920	KR045669
Cytospora gigalocus	CFCC 89621	*Juglans regia*	Qinghai, China	KR045629	KU710998	KU710958	KU710921	KR045670
Cytospora gigaspora	CFCC 50014	*Juniperus procumbens*	Shanxi, China	KR045630	KU710999.	KU710959	KU710922	KR045671
Cytospora gigaspora	**CFCC 89634[T]**	***Salix psammophila***	**Shaanxi, China**	**KF765671**	**KU711000**	**KU710960**	**KU710923**	**KR045672**
Cytospora globosa	MFLU 16-2054[T]	*Abies alba*	Italy	MT177935	NA	MT432212	MT454016	NA
Cytospora granati	CBS 144237[T]	*Punica granatum*	California, USA	MG971799	MG971949	NA	MG971514	MG971664
Cytospora haidianensis	CFCC 54056	*Euonymus alatus*	Beijing, China	MT360041	MT363978	MT363987	MT363997	MT364007
Cytospora haidianensis	CFCC 54057[T]	*Euonymus alatus*	Beijing, China	MT360042	MT363979	MT363988	MT363998	MT364008

Cytospora haidianensis	CFCC 54184	*Euonymus alatus*	Beijing, China	MT360043	MT363980	MT363989	MT363999	MT364009
Cytospora hippophaës	CFCC 89639	*Hippophaë rhamnoides*	Gansu, China	KR045632	KU711001	KU710961	KU710924	KR045673
Cytospora hippophaës	CFCC 89640	*Hippophaë rhamnoides*	Gansu, China	KF765682	KF765730	KU710962	KP310865	KR045674
Cytospora japonica	CFCC 89956	*Prunus cerasifera*	Ningxia, China	KR045624	KU710993	KU710953	KU710916	KR045665
Cytospora japonica	CFCC 89960	*Prunus cerasifera*	Ningxia, China	KR045625	KU710994	KU710954	KU710917	KR045666
Cytospora joaquinensis	CBS 144235	*Populus deltoides*	California, USA	MG971895	MG972044	NA	MG971605	MG971761
Cytospora junipericola	BBH 42444	*Juniperus communis*	Italy	MF190126	NA	NA	MF377579	NA
Cytospora junipericola	MFLU 17-0882^{T}	*Juniperus communis*	Italy	MF190125	NA	NA	MF377580	NA
Cytospora juniperina	CFCC 50501^{T}	*Juniperus przewalskii*	Sichuan, China	MH933632	MH933539	MH933602	MH933507	NA
Cytospora juniperina	CFCC 50502	*Juniperus przewalskii*	Sichuan, China	MH933633	MH933540	MH933603	MH933508	MH933572
Cytospora juniperina	CFCC 50503	*Juniperus przewalskii*	Sichuan, China	MH933634	MH933541	MH933604	MH933509	NA
Cytospora kantschavelii	CXY 1383	*Populus maximowiczii*	Jilin, China	KM034867	NA	NA	NA	NA
Cytospora kantschavelii	CXY 1386	*Populus maximowiczii*	Chongqing, China	KM034867	NA	NA	NA	NA
Cytospora kuanchengensis	CFCC 52464^{T}	*Castanea mollissima*	China	MK432616	MK442940	MK578076	NA	NA
Cytospora kuanchengensis	CFCC 52465	*Castanea mollissima*	China	MK432617	MK442941	MK578077	NA	NA
Cytospora longispora	CBS 144236^{T}	*Prunus domestica*	California, USA	MG971905	MG972054	NA	MG971615	MG971764
Cytospora longistiolata	MFLUCC 16-0628	*Salix × fragilis*	Russia	KY417734	KY417700	KY417802	NA	NA
Cytospora leucosperma	CFCC 89622	*Pyrus bretschneideri*	Gansu, China	KR045616	KU710988	KU710944	KU710911	KR045657
Cytospora leucosperma	CFCC 89894	*Pyrus bretschneideri*	Qinghai, China	KR045617	KU710989	KU710945	KU710912	KR045658
Cytospora leucostoma	CFCC 50023	*Cornus alba*	Shanxi, China	KR045635	KU711003	KU710964	KU710926	KR045676
Cytospora leucostoma	CFCC 50024	*Prunus pseudocerasus*	Qinghai, China	MH933640	MH933547	MH933605	NA	MH933576
Cytospora leucostoma	CFCC 53140	*Prunus sibirica*	Beijing, China	MN854445	MN850760	MN850746	MN850753	MN861115
Cytospora leucostoma	CFCC 53141	*Prunus sibirica*	Beijing, China	MN854446	MN850761	MN850747	MN850754	MN861116
Cytospora leucostoma	CFCC 53156	*Juglans mandshurica*	Beijing, China	MN854447	MN850762	MN850748	MN850755	MN861117

Cytospora leucostoma	CFCC 53167	*Prunus armeniaca*	Xinjiang, China	MK673056	MK673026	MK672998	MK672946	MK672972
Cytospora leucostoma	CFCC 53169	*Prunus persica*	Beijing, China	MK673080	MK673050	MK673020	MK672966	MK672996
Cytospora leucostoma	CFCC 53170	*Prunus persica*	Beijing, China	MK673081	MK673051	MK673021	MK672967	MK672997
Cytospora leucostoma	CFCC 54680	*Corylus heterophylla*	Beijing, China	MW839857	MW815941	MW815955	MW815890	MW883973
Cytospora leucostoma	CFCC 54681	*Corylus heterophylla*	Beijing, China	MW839857	MW815942	MW815956	MW815891	MW883974
Cytospora leucostoma	CFCC 54682	*Corylus heterophylla*	Beijing, China	MW839857	MW815943	MW815957	MW815892	MW883975
Cytospora leucostoma	CFCC 54683	*Corylus heterophylla*	Beijing, China	MW839857	MW815944	MW815958	MW815893	MW883976
Cytospora lumnitzericola	MFLUCC 17-0508[T]	*Lumnitzera racernosa*	Tailand	MG975778	MH253457	MH253453	NA	NA
Cytospora mali	CFCC 50028	*Malus pumila*	Gansu, China	MH933641	MH933548	MH933606	MH933513	MH933577
Cytospora mali	CFCC 50029	*Malus pumila*	Ningxia, China	MH933642	MH933549	MH933607	MH933514	MH933578
Cytospora mali	CFCC 50030	*Malus pumila*	Shaanxi, China	MH933643	MH933550	MH933608	MH933524	MH933579
Cytospora mali	CFCC 50031	*Crataegus* sp.	Shanxi, China	KR045636	KU711004	KU710965	KU710927	KR045677
Cytospora mali	CFCC 50044	*Malus baccata*	Qinghai, China	KR045637	KU711005	KU710966	KU710928	KR045678
Cytospora mali-spectabilis	CFCC 53181[T]	*Malus spectabilis* 'Royalty'	Xinjiang, China	MK673066	MK673036	MK673006	MK672953	MK672982
Cytospora melnikii	CFCC 89984	*Rhus typhina*	Xinjiang, China	MH933678	MH933551	MH933609	MH933515	MH933580
Cytospora melnikii	MFLUCC 15-0851	*Malus domestica*	Russia	KY417735	KY417701	KY417803	NA	NA
Cytospora melnikii	MFLUCC 16-0635	*Populus nigra* var. *italica*	Russia	KY417736	KY417702	KY417804	NA	NA
Cytospora myrtagena	CFCC 52454	*Castanea mollissima*	China	MK432614	MK442938	MK578074	NA	NA
Cytospora myrtagena	CFCC 52455	*Castanea mollissima*	China	MK432615	MK442939	MK578075	NA	NA
Cytospora nivea	MFLUCC 15-0860	*Salix acutifolia*	Russia	KY417737	KY417703	KY417805	NA	NA
Cytospora nivea	CFCC 89641	*Elaeagnus angustifolia*	Ningxia, China	KF765683	KU711006	KU710967	KU710929	KR045679
Cytospora nivea	**CFCC 89643**	***Salix psammophila***	**Shaanxi, China**	**KF765685**	**NA**	**KU710968**	**KP310863**	**KP310829**
Cytospora notastroma	NE_TFR5	*Populus tremuloides*	USA	JX438632	NA	NA	JX438543	NA
Cytospora notastroma	NE_TFR8	*Populus tremuloides*	USA	JX438633	NA	NA	JX438542	NA

Cytospora ochracea	CFCC 53164[T]	*Cotoneaster* sp.	Xinjiang, China	MK673060	MK673030	MK673001	MK672949	MK672976
Cytospora oleicola	CBS 144248[T]	*Olea europaea*	California, USA	MG971944	MG972098	NA	MG971660	MG971752
Cytospora olivacea	CFCC 53174	*Prunus cerasifera*	Xinjiang, China	MK673058	MK673028	MK672999	NA	MK672974
Cytospora olivacea	CFCC 53175	*Prunus dulcis*	Xinjiang, China	MK673062	MK673032	MK673003	NA	MK672978
Cytospora olivacea	CFCC 53176[T]	*Sorbus tianschanica*	Xinjiang, China	MK673068	MK673038	MK673008	MK672955	MK672984
Cytospora olivacea	CFCC 53177	*Prunus virginiana*	Xinjiang, China	MK673071	MK673041	MK673011	NA	MK672987
Cytospora palm	CXY 1276	*Cotinus coggygria*	Beijing, China	JN402990	NA	NA	KJ781296	NA
Cytospora palm	CXY 1280[T]	*Cotinus coggygria*	Beijing, China	JN411939	NA	NA	KJ781297	NA
Cytospora paracinnamomea	**CFCC 55452**	***Salix matsudana***	**Gansu, China**	**MZ702593**	**OK303455**	**OK303514**	**OK303575**	**OK303642**
Cytospora paracinnamomea	**CFCC 55453[T]**	***Salix matsudana***	**Gansu, China**	**MZ702594**	**OK303456**	**OK303515**	**OK303576**	**OK303643**
Cytospora paracinnamomea	**CFCC 55454**	***Salix matsudana***	**Gansu, China**	**MZ702597**	**OK303459**	**OK303518**	**OK303579**	**OK303646**
Cytospora paracinnamomea	**CFCC 55455[T]**	***Salix matsudana***	**Gansu, China**	**MZ702598**	**OK303460**	**OK303519**	**OK303580**	**OK303647**
Cytospora paracinnamomea	**CFCC 55456**	***Salix matsudana***	**Gansu, China**	**MZ702599**	**OK303461**	**OK303520**	**OK303581**	**OK303648**
Cytospora paracinnamomea	**CFCC 55457**	***Salix matsudana***	**Gansu, China**	**MZ702600**	**OK303462**	**OK303521**	**OK303582**	**OK303649**
Cytospora paracinnamomea	**CFCC 55458**	***Salix matsudana***	**Gansu, China**	**MZ702601**	**OK303463**	**OK303522**	**OK303583**	**OK303650**
Cytospora paracinnamomea	**CFCC 55459**	***Salix matsudana***	**Gansu, China**	**MZ702602**	**OK303464**	**OK303523**	**OK303584**	**OK303651**
Cytospora paracinnamomea	**CFCC 55460**	***Salix matsudana***	**Gansu, China**	**MZ702603**	**OK303465**	**OK303524**	**OK303585**	**OK303652**
Cytospora parakantschavelii	MFLUCC 15-0857[T]	*Populus × sibirica*	Russia	KY417738	KY417704	KY417806	NA	NA
Cytospora parakantschavelii	MFLUCC 16-0575	*Pyrus pyraster*	Russia	KY417739	KY417705	KY417807	NA	NA
Cytospora parapistaciae	CBS 144506[T]	*Pistacia vera*	California, USA	MG971804	MG971954	NA	MG971519	MG971669
Cytospora parasitica	MFLUCC 15-0507[T]	*Malus domestica*	Russia	KY417740	KY417706	KY417808	NA	NA
Cytospora parasitica	XJAU 2542-1	*Malus* sp.	Xinjiang, China	MH798884	NA	NA	MH813452	NA
Cytospora parasitica	CFCC 53171	*Malus pumila*	Xinjiang, China	MK673061	MK673031	MK673002	MK672950	MK672977
Cytospora parasitica	CFCC 53172	*Malus pumila*	Xinjiang, China	MK673069	MK673039	MK673009	MK672956	MK672985

Cytospora parasitica	CFCC 53173	*Berberis* sp.	Xinjiang, China	MK673070	MK673040	MK673010	MK672957	MK672986
Cytospora paratranslucens	MFLUCC 15-0506^{T}	*Populus alba* var. *bolleana*	Russia	KY417741	KY417707	KY417809	NA	NA
Cytospora paratranslucens	MFLUCC 16-0627	*Populus alba*	Russia	KY417742	KY417708	KY417810	NA	NA
Cytospora phialidica	MFLUCC 17-2498	*Alnus glutinosa*	Italy	MT177932	NA	MT432209	MT454014	NA
Cytospora piceae	CFCC 52841^{T*}	*Picea crassifolia*	Xinjiang, China	MH820398	MH820406	MH820395	MH820402	MH820387
Cytospora piceae	CFCC 52842*	*Picea crassifolia*	Xinjiang, China	MH820399	MH820407	MH820396	MH820403	MH820388
Cytospora pingbianensis	MFLUCC 18-1204^{T}	Undefined wood	Yunnan, China	MK912135	MN685817	MN685826	NA	NA
Cytospora pistaciae	CBS 144238^{T}	*Pistacia vera*	California, USA	MG971802	MG971952	NA	MG971517	MG971667
Cytospora platanicola	MFLU 17-0327	*Platanus hybrida*	Italy	MH253451	MH253449	MH253450	NA	NA
Cytospora platycladi	CFCC 50504^{T}*	*Platycladus orientalis*	Yunnan, China	MH933645	MH933552	MH933610	MH933516	MH933581
Cytospora platycladi	CFCC 50505*	*Platycladus orientalis*	Yunnan, China	MH933646	MH933553	MH933611	MH933517	MH933582
Cytospora platycladi	CFCC 50506*	*Platycladus orientalis*	Yunnan, China	MH933647	MH933554	MH933612	MH933518	MH933583
Cytospora platycladicola	CFCC 50038^{T*}	*Platycladus orientalis*	Gansu, China	KT222840	MH933555	MH933613	MH933519	MH933584
Cytospora platycladicola	CFCC 50039*	*Platycladus orientalis*	Gansu, China	KR045642	KU711008	KU710973	KU710931	KR045683
Cytospora plurivora	CBS 144239^{T}	*Olea europaea*	California, USA	MG971861	MG972010	NA	MG971572	MG971726
Cytospora populicola	CBS 144240	*Populus deltoides*	California, USA	MG971891	MG972040	NA	MG971601	MG971757
Cytospora populina	**CFCC 89644^{T}**	***Salix psammophila***	**Shaanxi, China**	**KF765686**	**KU711007**	**KU710969**	**KU710930**	**KR045681**
Cytospora populinopsis	CFCC 50032^{T}	*Sorbus aucuparia*	Ningxia, China	MH933648	MH933556	MH933614	MH933520	MH933585
Cytospora populinopsis	CFCC 50033	*Sorbus aucuparia*	Ningxia, China	MH933649	MH933557	MH933615	MH933521	MH933586
Cytospora predappioensis	MFLUCC 17-2458^{T}	*Platanus hybrida*	Italy	MG873484	NA	NA	NA	NA
Cytospora prunicola	MFLU 17-0995^{T}	*Prunus* sp.	Italy	MG742350	MG742353	MG742352	NA	NA
Cytospora pruni-mume	CFCC 53179	*Prunus armeniaca*	Xinjiang, China	MK673057	MK673027	NA	MK672947	MK672973
Cytospora pruni-mume	CFCC 53180^{T}	*Prunus mume*	Xinjiang, China	MK673067	MK673037	MK673007	MK672954	MK672983
Cytospora pruinopsis	CFCC 50034^{T}	*Ulmus pumila*	Shaanxi, China	KP281259	KP310836	KU710970	KP310849	KP310819

Cytospora pruinopsis	CFCC 50035	*Ulmus pumila*	Jilin, China	KP281260	KP310837	KU710971	KP310850	KP310820
Cytospora pruinopsis	CFCC 53153	*Ulmus pumila*	Beijing, China	MN854451	MN850763	MN850752	MN850759	MN861121
Cytospora pruinosa	CFCC 50036	*Syringa oblata*	Qinghai, China	KP310800	KP310832	NA	KP310845	KP310815
Cytospora pruinosa	CFCC 50037	*Syringa oblata*	Qinghai, China	MH933650	MH933558	NA	MH933522	MH933589
Cytospora pubescentis	MFLUCC 18-1201^{T}	*Quercus pubescens*	Forlì-Cesena, Italy	MK912130	MN685812	MN685821	NA	NA
Cytospora punicae	CBS 144244	*Punica granatum*	California, USA	MG971943	MG972091	NA	MG971654	MG971798
Cytospora quercicola	MFLU 17-0881	*Quercus* sp.	Italy	MF190128	NA	NA	NA	NA
Cytospora quercicola	MFLUCC 14-0867^{T}	*Quercus* sp.	Italy	MF190129	NA	NA	NA	NA
Cytospora ribis	CFCC 50026	*Ulmus pumila*	Qinghai, China	KP281267	KP310843	KU710972	KP310856	KP310826
Cytospora ribis	CFCC 50027	*Ulmus pumila*	Qinghai, China	KP281268	KP310844	NA	KP310857	KP310827
Cytospora rosae	MFLU 17-0885	*Rosa canina*	Italy	MF190131	NA	NA	NA	NA
Cytospora rosicola	CF 20197024^{T}	*Rosa* sp.	Tibet, China	MK673079	MK673049	MK673019	MK672965	MK672995
Cytospora rosigena	MFLUCC 18-0921^{T}	*Rosa* sp.	Russia	MN879872	NA	NA	NA	NA
Cytospora rostrata	**CFCC 89909**	***Salix cupularis***	**Gansu, China**	**KR045643**	**KU711009**	**KU710974**	**KU710932**	**KR045684**
Cytospora rostrata	**CFCC 89910**	***Salix cupularis***	**Gansu, China**	**KR045644**	**KU711010**	**KU710975**	**KU710933**	**NA**
Cytospora rusanovii	MFLUCC 15-0853	*Populus × sibirica*	Russia	KY417743	KY417709	KY417811	NA	NA
Cytospora rusanovii	MFLUCC 15-0854^{T}	*Salix babylonica*	Russia	KY417744	KY417710	KY417812	NA	NA
Cytospora salicacearum	MFLUCC 15-0509	*Salix alba*	Russia	KY417746	KY417712	KY417814	NA	NA
Cytospora salicacearum	MFLUCC 15-0861	*Salix × fragilis*	Russia	KY417745	KY417711	KY417813	NA	NA
Cytospora salicacearum	MFLUCC 16-0587	*Prunus cerasus*	Russia	KY417742	KY417708	KY417810	NA	NA
Cytospora salicacearum	MFLUCC 16-0576	*Populus nigra* var. *italica*	Russia	KY417741	KY417707	KY417809	NA	NA
Cytospora salicicola	MFLUCC 14-1052^{T}	*Salix alba*	Russia	KU982636	KU982637	NA	NA	NA
Cytospora salicicola	MFLUCC 15-0866	*Salix* sp.	Thailand	KY417749	KY417715	KY417817	NA	NA
Cytospora salicina	MFLUCC 15-0862	*Salix alba*	Russia	KY417750	KY417716	KY417818	NA	NA

Cytospora salicina	MFLUCC 16-0637	*Salix × fragilis*	Russia	KY417751	KY417717	KY417819	NA	NA
Cytospora schulzeri	CFCC 50042	*Malus pumila*	Gansu, China	KR045650	KU711014	KU710981	KU710937	KR045691
Cytospora sibiraeae	CFCC 50045^{T}	*Sibiraea angustata*	Gansu, China	KR045651	KU711015	KU710982	KU710938	KR045692
Cytospora sibiraeae	CFCC 50046	*Sibiraea angustata*	Gansu, China	KR045652	KU711015	KU710983	KU710939	KR045693
Cytospora sophorae	CFCC 50047	*Styphnolobium japonicum*	Shanxi, China	KR045653	KU711017	KU710984	KU710940	KR045694
Cytospora sophorae	CFCC 50048	*Magnolia grandiflora*	Shanxi, China	MH820401	MH820409	MH820397	MH820405	MH820390
Cytospora sophorae	CFCC 89598	*Styphnolobium japonicum*	Gansu, China	KR045654	KU711018	KU710985	KU710941	KR045695
Cytospora sophoricola	CFCC 89596	*Styphnolobium japonicum* var. *pendula*	Gansu, China	KR045656	KU711020	KU710987	KU710943	KR045697
Cytospora sophoricola	CFCC 89595^{T}	*Styphnolobium japonicum* var. *pendula*	Gansu, China	KR045655	KU711019	KU710986	KU710942	KR045696
Cytospora sophoriopsis	**CFCC 55469**	***Salix matsudana***	**Gansu, China**	**MZ702583**	**OK303445**	**OK303504**	**OK303565**	**OK303632**
Cytospora sophoriopsis	CFCC 89600	*Styphnolobium japonicum*	Gansu, China	KR045623	KU710992	KU710951	KU710915	KP310817
Cytospora sorbi	MFLUCC 16-0631^{T}	*Sorbus aucuparia*	Russia	KY417752	KY417718	KY417820	NA	NA
Cytospora sorbicola	MFLUCC 16-0584^{T}	*Acer pseudoplatanus*	Russia	KY417755	KY417721	KY417823	NA	NA
Cytospora sorbicola	MFLUCC 16-0633	*Cotoneaster melanocarpus*	Russia	KY417758	KY417724	KY417826	NA	NA
Cytospora sorbina	CF 20197660^{T}	*Sorbus tianschanica*	Xinjiang, China	MK673052	MK673022	NA	MK672943	MK672968
Cytospora spiraeae	CFCC 50049^{T}	*Spiraea salicifolia*	Gansu, China	MG707859	MG708196	MG708199	NA	NA
Cytospora spiraeae	CFCC 50050	*Spiraea salicifolia*	Gansu, China	MG707860	MG708197	MG708200	NA	NA
Cytospora spiraeicola	CFCC 53138^{T}	*Spiraea salicifolia*	Beijing, China	MN854448	NA	MN850749	MN850756	MN861118
Cytospora spiraeicola	CFCC 53139	*Tilia nobilis*	Beijing, China	MN854449	NA	MN850750	MN850757	MN861119
Cytospora tamaricicola	CFCC 50507	*Rosa multifolora*	Yunnan, China	MH933651	MH933559	MH933616	MH933525	MH933587
Cytospora tamaricicola	CFCC 50508^{T}	*Tamarix chinensis*	Yunnan, China	MH933652	MH933560	MH933617	MH933523	MH933588
Cytospora tanaitica	MFLUCC 14-1057^{T}	*Betula pubescens*	Russia	KT459411	KT459413	NA	NA	NA

Cytospora thailandica	MFLUCC 17-0262[T]	*Xylocarpus moluccensis*	Thailand	MG975776	MH253459	MH253455	NA	NA
Cytospora thailandica	MFLUCC 17-0263[T]	*Xylocarpus moluccensis*	Thailand	MG975777	MH253460	MH253456	NA	NA
Cytospora tibetensis	CF 20197026	*Cotoneaster* sp.	Tibet, China	MK673076	MK673046	MK673016	MK672962	MK672992
Cytospora tibetensis	CF 20197029	*Cotoneaster* sp.	Tibet, China	MK673077	MK673047	MK673017	MK672963	MK672993
Cytospora tibetensis	CF 20197032[T]	*Cotoneaster* sp.	Tibet, China	MK673078	MK673048	MK673018	MK672964	MK672994
Cytospora tibouchinae	CPC 26333[T]	*Tibouchina semidecandra*	France	KX228284	NA	NA	NA	NA
Cytospora translucens	CXY 1351	*Populus davidiana*	Inner Mongolia, China	KM034874	NA	NA	NA	KM034895
Cytospora translucens	CXY 1359	*Populus* × Beijingensis	Beijing, China	KM034871	NA	NA	NA	KM034894
Cytospora ulmi	MFLUCC 15-0863[T]	*Ulmus minor*	Russia	KY417759	NA	NA	NA	NA
Cytospora verrucosa	CFCC 53157 [T]	*Platycladus orientalis*	Beijing, China	MW418408	NA	MW422911	MW422923	MW422935
Cytospora verrucosa	CFCC 53158	*Platycladus orientalis*	Beijing, China	MW418410	MW422901	MW422913	MW422925	MW422937
Cytospora verrucosa	CFCC 54369	*Platycladus orientalis*	Beijing, China	MW418409	NA	MW422912	MW422924	MW422936
Cytospora verrucosa	CFCC 54370	*Platycladus orientalis*	Beijing, China	MW418411	MW422902	MW422914	MW422926	MW422938
Cytospora vinacea	CBS 141585[T]	*Vitis interspecific* hybrid 'Vidal'	USA	KX256256	NA	NA	KX256277	KX256235
Cytospora viridistroma	CBS 202.36[T]	*Cercis canadensis* Castigl.	USA	MN172408	NA	NA	MN271853	NA
Cytospora viticola	Cyt2	*Vitis interspecific* hybrid 'Frontenac'	USA	KX256238	NA	NA	KX256259	KX256217
Cytospora viticola	CBS 141586[T]	*Vitis vinifera* 'CabernetFranc'	USA	KX256239	NA	NA	KX256260	KX256218
Cytospora xinjiangensis	CFCC 53182	*Rosa* sp.	Xinjiang, China	MK673064	MK673034	MK673004	MK672951	MK672980
Cytospora xinjiangensis	CFCC 53183[T]	*Rosa* sp.	Xinjiang, China	MK673065	MK673035	MK673005	MK672952	MK672981
Cytospora xinglongensis	CFCC 52458	*Castanea mollissima*	China	MK432622	MK442946	MK578082	NA	NA

Cytospora xinglongensis	CFCC 52459	*Castanea mollissima*	China	MK432623	MK442947	MK578083	NA	NA
Cytospora xylocarpi	MFLUCC 17-0251[T]	*Xylocarpus granatum*	Thailand	MG975775	MH253458	MH253454	NA	NA
Diaporthe vaccinii	CBS 160.32	*Vaccinium macrocarpon*	USA	KC343228	JQ807297	NA	KC343954	KC344196

[1] Acronyms: ATCC: American Type Culture Collecton, Virginia, USA; BBH: BIOTEC Bangkok Herbarium, National Science and Technology Development Agency, Thailand; CBS: Westerdijk Fungal Biodiversity Institute (CBS-KNAW Fungal Biodiversity Centre), Utrecht, The Netherlands; CFCC: China Forestry Culture Collection Centre, Beijing, China; CMW: Culture collection of Michael Wingfield, University of Pretoria, South Africa; CPC: Culture collection of Pedro Crous, The Netherlands; IMI: Culture collection of the International Mycological Institute, CABI Bioscience, Egham, Surrey, UK; MFLU: Mae Fah Luang University herbarium, Thailand; MFLUCC: Mae Fah Luang University Culture Collection, Thailand; MUCC: Murdoch University Culture Collection, Perth, Australia; NE: Gerard Adams collections, University of Nebraska, Lincoln NE, USA; PPRI: Culture collection of the Plant Protection Research Institute, Agriculture Research Center, Pretoria, South Africa; XJAU: Xinjiang Agricultural University, Xinjiang, China; NA: not applicable. All the new isolates used in this study are in bold and the type materials are marked with T.

Table S3. The regression equations and the estimated optimum growth temperature, pH value, and carbon source.

Species	Regression Equations of Temperature (x = temperature (°C), y = growth (colony diameter, mm))	Temperature Range (°C)	Estimated Optimum Growth Temperature	Regression Equationsof pH (x = pH, y = growth (colony diameter, mm))	pH Range	Estimated Optimum Growth pH	Estimated Optimum Growth Carbon Source
Cytospora alba (CFCC 55402)	$y_{Day1} = -0.0005x^3 + 0.0035x^2 + 0.6438x + 4.6768$ ($R^2 = 0.7348$)<br>$y_{Day2} = -0.0009x^3 - 0.033x^2 + 2.6289x + 2.3212$ ($R^2 = 0.8171$)<br>$y_{Day3} = -0.0007x^3 - 0.1103x^2 + 5.3437x + 1.9394$ ($R^2 = 0.8513$)<br>$y_{Day4} = -0.0004x^3 - 0.1762x^2 + 7.4157x + 7.4949$ ($R^2 = 0.9055$)	0–30	21.1	$y_{Day1} = 0.3546x^3 - 8.5492x^2 + 61.603x - 110.27$ ($R^2 = 0.9957$)<br>$y_{Day2} = 0.5799x^3 - 14.068x^2 + 101.43x - 177.67$ ($R^2 = 0.9252$)<br>$y_{Day3} = 0.6425x^3 - 16.478x^2 + 124.19x - 217.02$ ($R^2 = 0.8945$)<br>$y_{Day4} = 0.4139x^3 - 11.886x^2 + 95.703x - 151.21$ ($R^2 = 0.8693$)	3.0–10.0	5.6	fructose
Cytospora paracinnamomea (CFCC 55453)	$y_{Day1} = -0.0006x^3 + 0.0147x^2 + 0.3385x + 5.5222$ ($R^2 = 0.8937$)<br>$y_{Day2} = -0.0009x^3 - 0.0406x^2 + 3.0735x + 2.7606$ ($R^2 = 0.9161$)<br>$y_{Day3} = -0.0017x^3 - 0.0843x^2 + 5.8622x + 5.7758$ ($R^2 = 0.944$)<br>$y_{Day4} = -0.0014x^3 - 0.1473x^2 + 7.7947x + 13.211$ ($R^2 = 0.9641$)	0–30	21.9	$y_{Day1} = 0.2383x^3 - 5.3296x^2 + 35.599x - 57.712$ ($R^2 = 0.9028$)<br>$y_{Day2} = 0.9923x^3 - 23.044x^2 + 160.21x - 287.09$ ($R^2 = 0.9761$)<br>$y_{Day3} = 1.0142x^3 - 25.35x^2 + 188.44x - 350.98$ ($R^2 = 0.9806$)<br>$y_{Day4} = 1.037x^3 - 27.082x^2 + 208.41x - 391.11$ ($R^2 = 0.9361$)	3.0–10.0	5.4	fructose, glucose, and maltose
