# Supplementary figures and images for: Fungal Richness of Cytospora Species Associated with Willow Canker Disease in China
Source: J Fungi (Basel). 2022 Apr 7;8(4):377. doi: 10.3390/jof8040377 (PMC9030772; doi:10.3390/jof8040377)

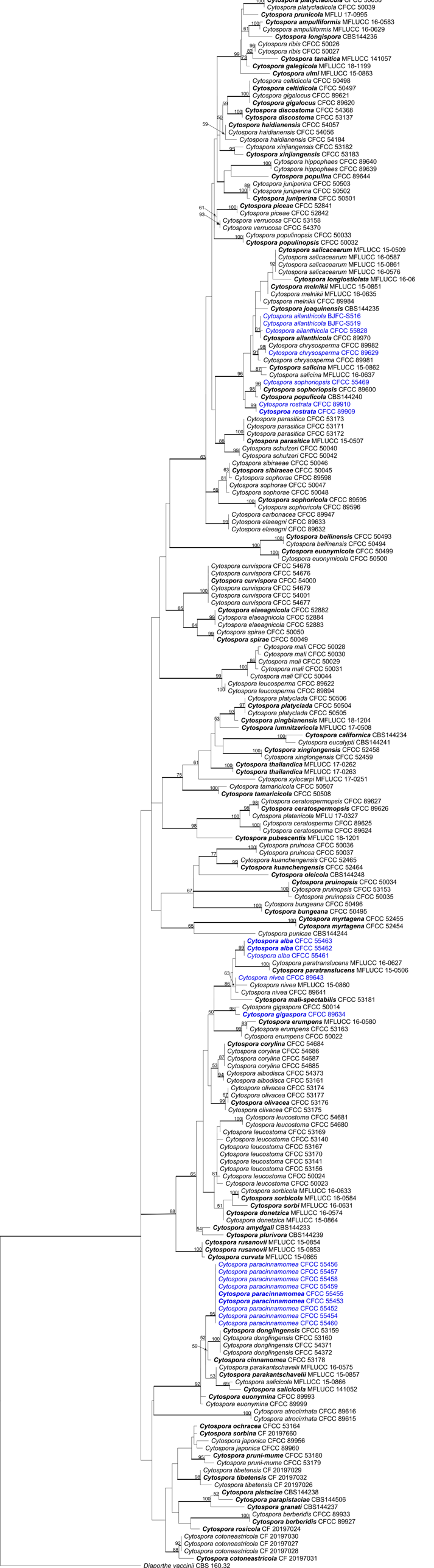

Supplement: Supplementary file 1 [file jof-08-00377-s001.zip › Figure S2.pdf]

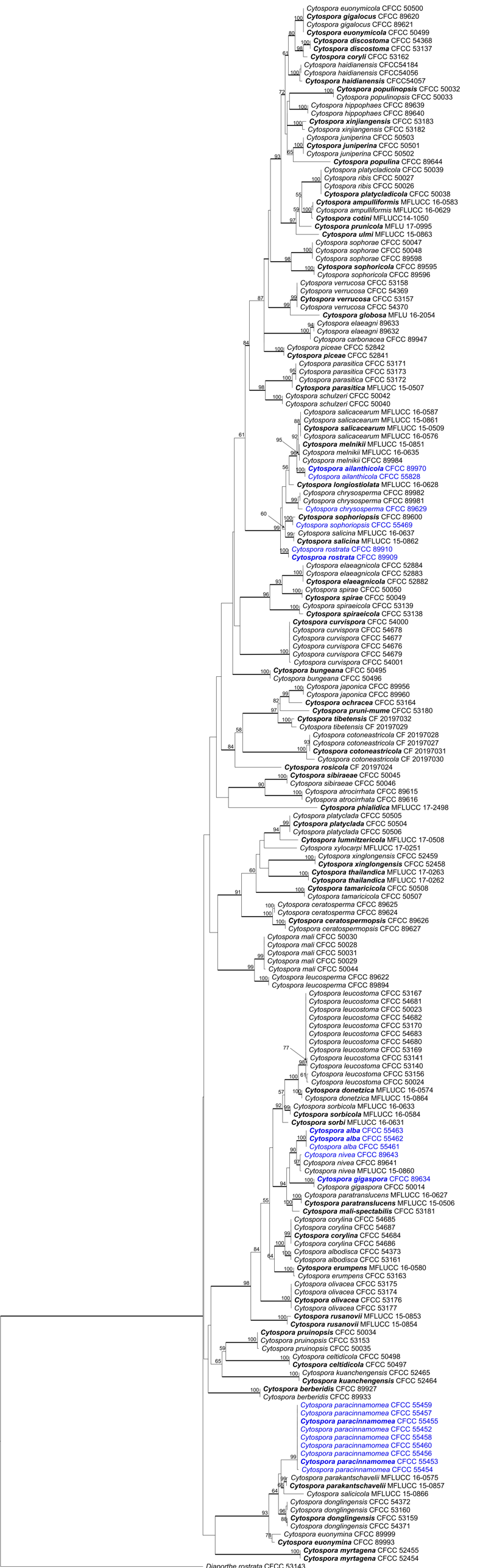

Supplement: Supplementary file 1 [file jof-08-00377-s001.zip › Figure S3.pdf]

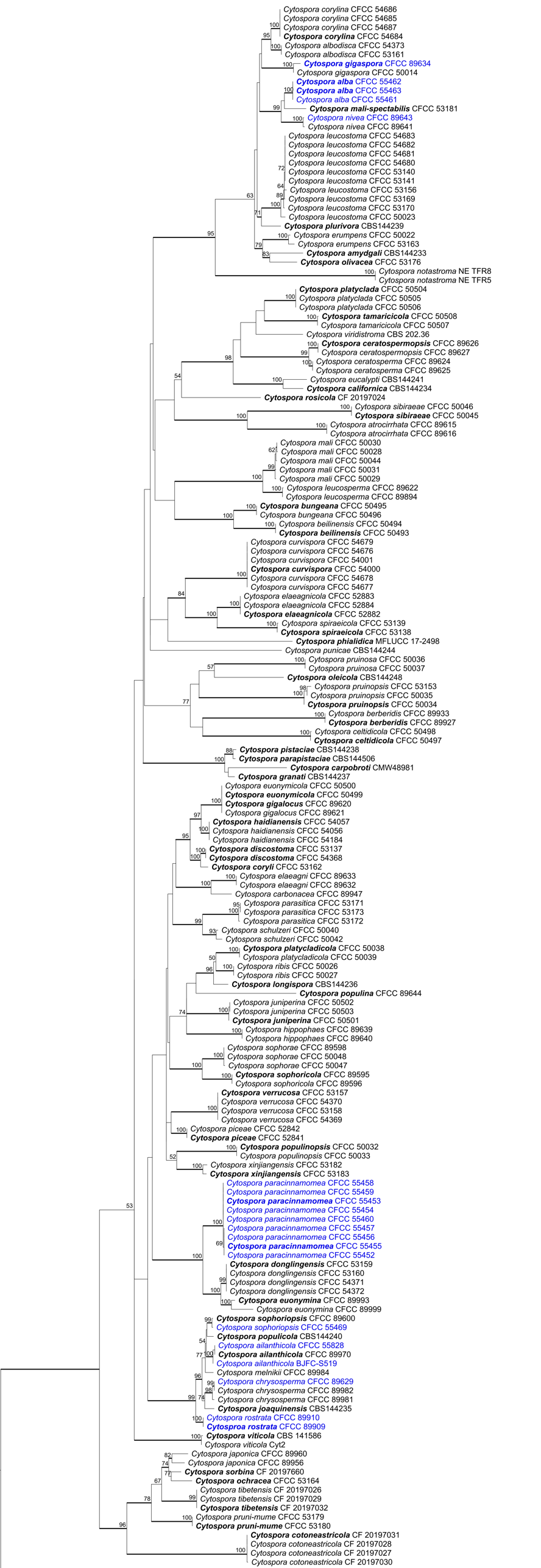

Supplement: Supplementary file 1 [file jof-08-00377-s001.zip › Figure S4.pdf]

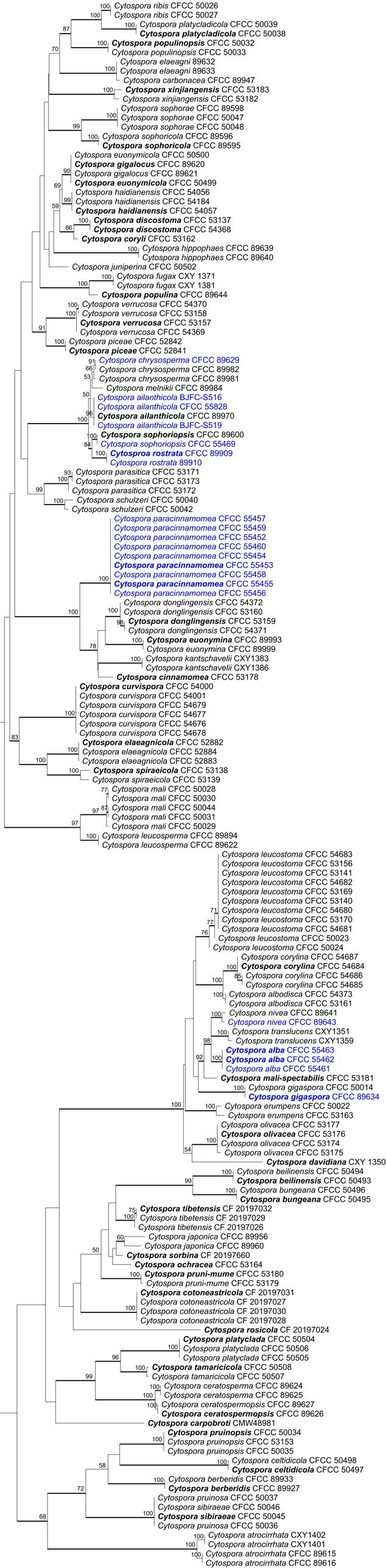

*Diaporthe vaccinii* CBS 160.32

Supplement: Supplementary file 1 [file jof-08-00377-s001.zip › Figure S5.pdf]
