# Supplementary material for: Fungal Richness of Cytospora Species Associated with Willow Canker Disease in China
Source: J Fungi (Basel). 2022 Apr 7;8(4):377. doi: 10.3390/jof8040377 (PMC9030772; doi:10.3390/jof8040377)
Supplement: Supplementary file 1 [file Figure S1.pdf › Figure S1.pdf]

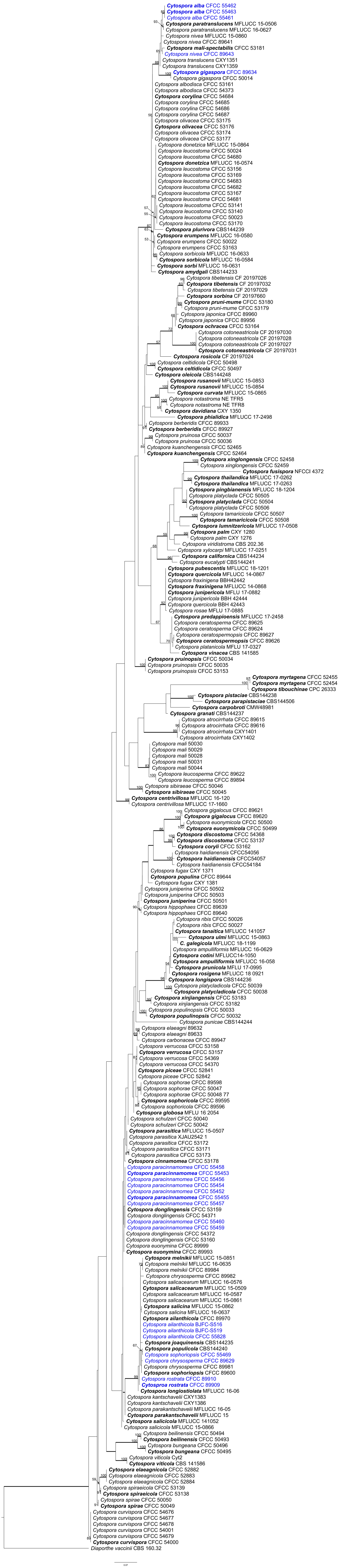
Cytospora alba CFCC 55462
Cytospora alba CFCC 55463
Cytospora alba CFCC 55461
Cytospora paratranslucens MFLUCC 15-0506
Cytospora paratranslucens MFLUCC 16-0627
Cytospora nivea MFLUCC 15-0860
Cytospora nivea CFCC 89641
Cytospora mali-spectabilis CFCC 53181
Cytospora nivea CFCC 89643
Cytospora translucens CXY1351
Cytospora translucens CXY1359
Cytospora gigaspora CFCC 89634
Cytospora gigaspora CFCC 50014
Cytospora albodisca CFCC 53161
Cytospora albodisca CFCC 54373
Cytospora corylina CFCC 54684
Cytospora corylina CFCC 54685
Cytospora corylina CFCC 54686
Cytospora corylina CFCC 54687
Cytospora olivacea CFCC 53175
Cytospora olivacea CFCC 53176
Cytospora olivacea CFCC 53174
Cytospora olivacea CFCC 53177
Cytospora donetzica MFLUCC 15-0864
Cytospora leucostoma CFCC 50024
Cytospora leucostoma CFCC 54680
Cytospora donetzica MFLUCC 16-0574
Cytospora leucostoma CFCC 53156
Cytospora leucostoma CFCC 53169
Cytospora leucostoma CFCC 54683
Cytospora leucostoma CFCC 54682
Cytospora leucostoma CFCC 53167
Cytospora leucostoma CFCC 54681
Cytospora leucostoma CFCC 53141
Cytospora leucostoma CFCC 53140
Cytospora leucostoma CFCC 50023
Cytospora leucostoma CFCC 53170
Cytospora plurivora CBS144239
Cytospora erumpens MFLUCC 16-0580
Cytospora erumpens CFCC 50022
Cytospora erumpens CFCC 53163
Cytospora sorbicola MFLUCC 16-0633
Cytospora sorbicola MFLUCC 16-0584
Cytospora sorbi MFLUCC 16-0631
Cytospora amygdali CBS144233
Cytospora tibetensis CF 20197026
Cytospora tibetensis CF 20197032
Cytospora tibetensis CF 20197029
Cytospora sorbina CF 20197660
Cytospora pruni-mume CFCC 53180
Cytospora pruni-mume CFCC 53179
Cytospora japonica CFCC 89960
Cytospora japonica CFCC 89956
Cytospora ochracea CFCC 53164
Cytospora cotoneastricola CF 20197030
Cytospora cotoneastricola CF 20197028
Cytospora cotoneastricola CF 20197027
Cytospora cotoneastricola CF 20197031
Cytospora rosicola CF 20197024
Cytospora celtidicola CFCC 50498
Cytospora celtidicola CFCC 50497
Cytospora oleicola CBS144248
Cytospora rusanovii MFLUCC 15-0853
Cytospora rusanovii MFLUCC 15-0854
Cytospora curvata MFLUCC 15-0865
Cytospora notastroma NE TFR5
Cytospora notastroma NE TFR8
Cytospora davidiana CXY 1350
Cytospora phialidica MFLUCC 17-2498
Cytospora berberidis CFCC 89933
Cytospora berberidis CFCC 89927
Cytospora pruinosa CFCC 50037
Cytospora pruinosa CFCC 50036
Cytospora kuanchengensis CFCC 52465
Cytospora kuanchengensis CFCC 52464
Cytospora xinglongensis CFCC 52458
Cytospora xinglongensis CFCC 52459
Cytospora fusispora NFCCI 4372
Cytospora thailandica MFLUCC 17-0262
Cytospora thailandica MFLUCC 17-0263
Cytospora pingbianensis MFLUCC 18-1204
Cytospora platyclada CFCC 50505
Cytospora platyclada CFCC 50504
Cytospora platyclada CFCC 50506
Cytospora tamaricicola CFCC 50507
Cytospora tamaricicola CFCC 50508
Cytospora lumnitzericola MFLUCC 17-0508
Cytospora palm CXY 1280
Cytospora palm CXY 1276
Cytospora viridistroma CBS 202.36
Cytospora xylocarpi MFLUCC 17-0251
Cytospora californica CBS144234
Cytospora eucalypti CBS144241
Cytospora pubescentis MFLUCC 18-1201
Cytospora quercicola MFLUCC 14-0867
Cytospora fraxinigena BBH42442
Cytospora fraxinigena MFLUCC 14-0868
Cytospora junipericola MFLU 17-0882
Cytospora junipericola BBH 42444
Cytospora quercicola BBH 42443
Cytospora rosae MFLU 17-0885
Cytospora predappioensis MFLUCC 17-2458
Cytospora ceratosperma CFCC 89625
Cytospora ceratosperma CFCC 89624
Cytospora ceratospermopsis CFCC 89627
Cytospora ceratospermopsis CFCC 89626
Cytospora platanicola MFLU 17-0327
Cytospora vinacea CBS 141585
Cytospora pruinopsis CFCC 50034
Cytospora pruinopsis CFCC 50035
Cytospora pruinopsis CFCC 53153
Cytospora myrtagena CFCC 52455
Cytospora myrtagena CFCC 52454
Cytospora tibouchinae CPC 26333
Cytospora pistaciae CBS144238
Cytospora parapistaciae CBS144506
Cytospora carpobroti CMW48981
Cytospora granati CBS144237
Cytospora atrocirrhata CFCC 89615
Cytospora atrocirrhata CFCC 89616
Cytospora atrocirrhata CXY1401
Cytospora atrocirrhata CXY1402
Cytospora mali 50030
Cytospora mali 50029
Cytospora mali 50028
Cytospora mali 50031
Cytospora mali 50044
Cytospora leucosperma CFCC 89622
Cytospora leucosperma CFCC 89894
Cytospora sibiraeae CFCC 50046
Cytospora sibiraeae CFCC 50045
Cytospora centrivillosa MFLUCC 16-120
Cytospora centrivillosa MFLUCC 17-1660
Cytospora gigalocus CFCC 89621
Cytospora gigalocus CFCC 89620
Cytospora euonymicola CFCC 50500
Cytospora euonymicola CFCC 50499
Cytospora discotoma CFCC 54368
Cytospora discotoma CFCC 53137
Cytospora coryli CFCC 53162
Cytospora haidianensis CFCC54056
Cytospora haidianensis CFCC54057
Cytospora haidianensis CFCC54184
Cytospora fugax CXY 1371
Cytospora populina CFCC 89644
Cytospora fugax CXY 1381
Cytospora juniperina CFCC 50502
Cytospora juniperina CFCC 50503
Cytospora juniperina CFCC 50501
Cytospora hippophaes CFCC 89639
Cytospora hippophaes CFCC 89640
Cytospora ribis CFCC 50026
Cytospora ribis CFCC 50027
Cytospora tanaitica MFLUCC 141057
Cytospora ulmi MFLUCC 15-0863
C. galegicola MFLUCC 18-1199
Cytospora ampulliformis MFLUCC 16-0629
Cytospora cotini MFLUCC14-1050
Cytospora ampulliformis MFLUCC 16-058
Cytospora prunicola MFLU 17-0995
Cytospora rosigena MFLUCC 18 0921
Cytospora longispora CBS144236
Cytospora platycladicola CFCC 50039
Cytospora platycladicola CFCC 50038
Cytospora xinjiangensis CFCC 53183
Cytospora xinjiangensis CFCC 53182
Cytospora populinopsis CFCC 50033
Cytospora populinopsis CFCC 50032
Cytospora punicae CBS144244
Cytospora elaeagni 89632
Cytospora elaeagni 89633
Cytospora carbonacea CFCC 89947
Cytospora verrucosa CFCC 53158
Cytospora verrucosa CFCC 53157
Cytospora verrucosa CFCC 54369
Cytospora verrucosa CFCC 54370
Cytospora piceae CFCC 52841
Cytospora piceae CFCC 52842
Cytospora sophorae CFCC 89598
Cytospora sophorae CFCC 50047
Cytospora sophorae CFCC 50048 77
Cytospora sophoricola CFCC 89595
Cytospora sophoricola CFCC 89596
Cytospora globosa MFLU 16 2054
Cytospora schulzeri CFCC 50040
Cytospora schulzeri CFCC 50042
Cytospora parasitica MFLUCC 15-0507
Cytospora parasitica XJAU2542 1
Cytospora parasitica CFCC 53172
Cytospora parasitica CFCC 53171
Cytospora parasitica CFCC 53173
Cytospora cinnamomea CFCC 53178
Cytospora paracinnamomea CFCC 55458
Cytospora paracinnamomea CFCC 55453
Cytospora paracinnamomea CFCC 55456
Cytospora paracinnamomea CFCC 55454
Cytospora paracinnamomea CFCC 55452
Cytospora paracinnamomea CFCC 55455
Cytospora paracinnamomea CFCC 55457
Cytospora donglingensis CFCC 53159
Cytospora donglingensis CFCC 54371
Cytospora paracinnamomea CFCC 55460
Cytospora paracinnamomea CFCC 55459
Cytospora donglingensis CFCC 54372
Cytospora donglingensis CFCC 53160
Cytospora euonymina CFCC 89999
Cytospora euonymina CFCC 89993
Cytospora melnikii MFLUCC 15-0851
Cytospora melnikii MFLUCC 16-0635
Cytospora melnikii CFCC 89984
Cytospora chrysosperma CFCC 89982
Cytospora salicacearum MFLUCC 16-0576
Cytospora salicacearum MFLUCC 15-0509
Cytospora salicacearum MFLUCC 16-0587
Cytospora salicacearum MFLUCC 15-0861
Cytospora salicina MFLUCC 15-0862
Cytospora salicina MFLUCC 16-0637
Cytospora ailanthicola CFCC 89970
Cytospora ailanthicola BJFC-S516
Cytospora ailanthicola BJFC-S519
Cytospora ailanthicola CFCC 55828
Cytospora joaquinensis CBS144235
Cytospora populicola CBS144240
Cytospora sophoriopsis CFCC 55469
Cytospora chrysosperma CFCC 89629
Cytospora chrysosperma CFCC 89981
Cytospora sophoriopsis CFCC 89600
Cytospora rostrata CFCC 89910
Cytospora rostrata CFCC 89909
Cytospora longiostiolata MFLUCC 16-06
Cytospora kantschavelii CXY1383
Cytospora kantschavelii CXY1386
Cytospora parakantschavelii MFLUCC 16-05
Cytospora parakantschavelii MFLUCC 15
Cytospora salicicola MFLUCC 141052
Cytospora salicicola MFLUCC 15-0866
Cytospora beilinensis CFCC 50494
Cytospora beilinensis CFCC 50493
Cytospora bungeana CFCC 50496
Cytospora bungeana CFCC 50495
Cytospora viticola Cyt2
Cytospora viticola CBS 141586
Cytospora elaeagnicola CFCC 52882
Cytospora elaeagnicola CFCC 52883
Cytospora elaeagnicola CFCC 52884
Cytospora spiraeicola CFCC 53139
Cytospora spiraeicola CFCC 53138
Cytospora spirae CFCC 50050
Cytospora spirae CFCC 50049
Cytospora curvispora CFCC 54676
Cytospora curvispora CFCC 54677
Cytospora curvispora CFCC 54678
Cytospora curvispora CFCC 54001
Cytospora curvispora CFCC 54679
Cytospora curvispora CFCC 54000
Diaporthe vaccinii CBS 160.32
